# Catechol siderophores framed on 2,3-dihydroxybenzoyl-L-serine from *Streptomyces varsoviensis*

**DOI:** 10.3389/fmicb.2023.1182449

**Published:** 2023-05-03

**Authors:** Zhixiang Liu, Tingting Huang, Qing Shi, Zixin Deng, Shuangjun Lin

**Affiliations:** ^1^State Key Laboratory of Microbial Metabolism, Joint International Research Laboratory on Metabolic & Developmental Sciences, School of Life Sciences & Biotechnology, Shanghai Jiao Tong University, Shanghai, China; ^2^Haihe Laboratory of Synthetic Biology, Tianjin, China; ^3^Frontiers Science Center for Transformative Molecules, Shanghai Jiao Tong University, Shanghai, China

**Keywords:** catecholate, siderophore, enterobactin, streptomyces, culture media, NRPS

## Abstract

Enterobactin is an archetypical catecholate siderophore that plays a key role in the acquisition of ferric iron by microorganisms. Catechol moieties have been shown to be promising siderophore cores. Variants of the conserved 2,3-dihydroxybenzoate (DHB) moiety with structural modifications expand the bioactivity. *Streptomyces* are characterized by metabolites with diverse structures. The genomic sequence of *Streptomyces varsoviensis* indicated that it possessed a biosynthetic gene cluster for DHB containing siderophores and metabolic profiling revealed metabolites correlated with catechol-type natural products. Here, we report the discovery of a series of catecholate siderophores produced by *S. varsoviensis* and a scale-up fermentation was performed to purify these compounds for structural elucidation. A biosynthetic route for the catecholate siderophores is also proposed. These new structural features enrich the structural diversity of the enterobactin family compounds. One of the new linear enterobactin congeners shows moderate activity against a food-borne pathogen *Listeria monocytogenes*. This work demonstrated that changing culture conditions is still a promising approach to explore unexplored chemical diversity. The availability of the biosynthetic machinery will enrich the genetic toolbox of catechol siderophores and facilitate such engineering efforts.

## 1. Introduction

As an essential metal for microbial growth, iron possesses the ability to facilitate electron transport across a range of reduction–oxidation potentials. It also functions as an important cofactor for enzymes involved in a wide range of important cellular processes, such as cell proliferation, DNA synthesis and protection from reactive oxygen species (Hider and Kong, 2010). However, in most natural environments, the chemical activity of accessible ferric iron is extremely limited, counteracting its biological utilization. To overcome this problem, the intelligent microorganisms develop a siderophore-mediated iron acquisition strategy by producing iron-chelating molecules known as siderophores, which solubilize Fe^3+^ for microbial utilization and facilitate bacterial colonization or infection of eukaryotic hosts by releasing host iron for bacterial uptake (Miethke and Marahiel, 2007). In addition, siderophores are known to be an important virulence determinant for many pathogenic microbes.

In nature, siderophore-producing bacteria can secrete a large group of structurally diverse iron chelators. According to the chemical architecture of the iron coordinating moieties, siderophores can be classified as catecholate (e.g., enterobactin, cacillibactin), hydroxamate (e.g., coelichelin), phenolate, carboxylate, and mixed type (e.g., yersiniabactin, pyochelin) (Miethke and Marahiel, 2007). Since these chelating moieties have various affinities toward iron, a more plausible explanation for the evolution of different types of siderophores would be the intense competition between different microbial species or between microbes and their hosts (Hider and Kong, 2010).

**Figure 1:**
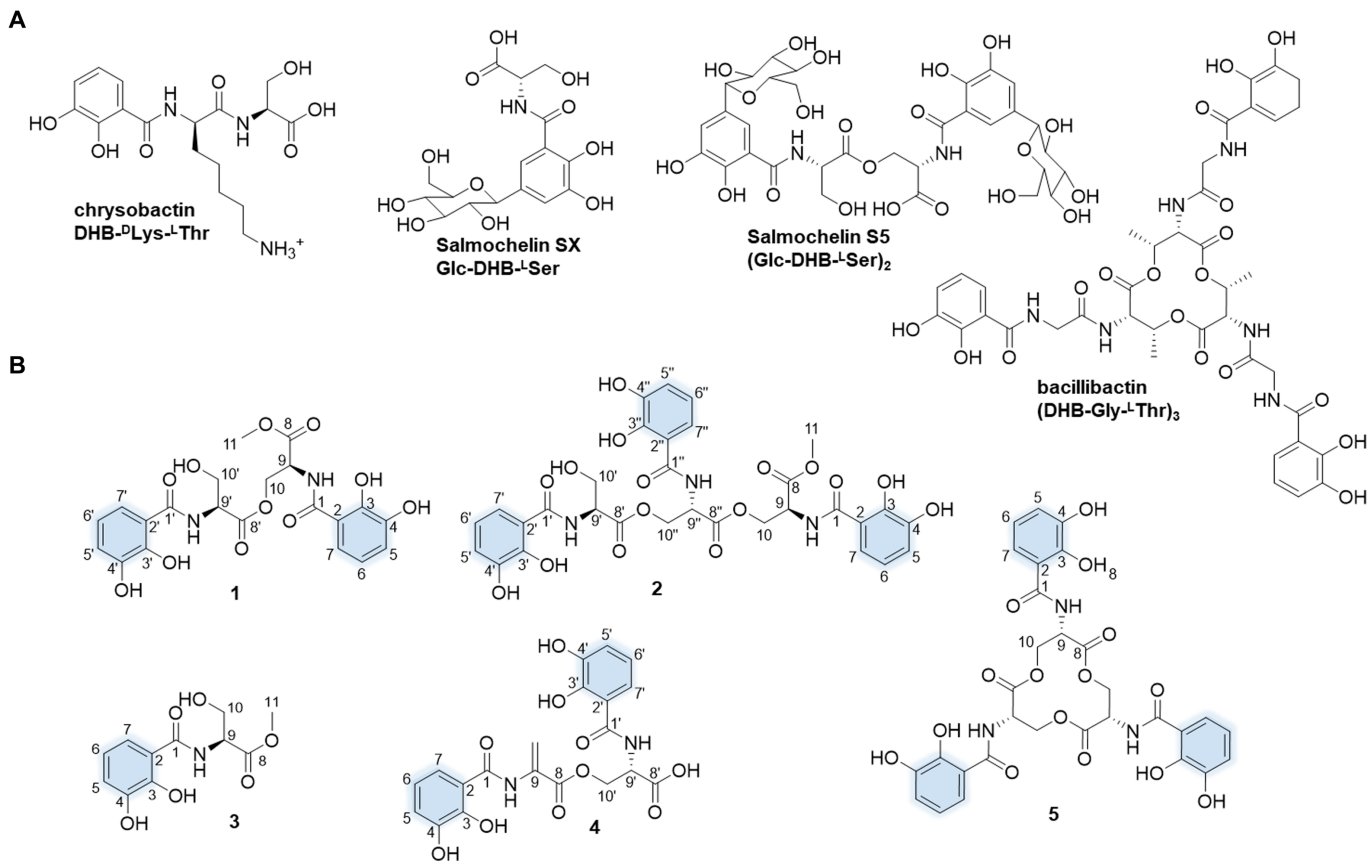
Molecular structures of the presentative catechol siderophores **(A)** and isolated siderophores in this study. The iron-coordinating moieties are indicated in blue **(B)**.

Of these siderophore metabolites, the catechol siderophores are attractive because of their extremely high affinity for Fe^3+^, due to the presence of the 2,3-dihydroxybenzamide motifs. Enterobactin is a remarkable tri-catechol siderophore with an exceptionally high affinity for Fe^3+^ (*K*_a_ = 10^52^), which is the highest affinity for iron reported to date (Raymond et al., 2003; Khan et al., 2018). Enterobactin ligands coordinate iron through 2,3-catecholate moieties, which are attached to three L-serine residues via amide bonds in a tri-lactone macrocycle (Figure 1). As it is naturally produced by certain Gram-negative Enterobacteriaceae such as *Escherichia coli* and *Salmonella typhimurium*, the electrochemical fingerprint of enterobactin has been well studied and provides an opportunity for rapid detection of bacterial contamination (Yancey et al., 1979; Bergstrom et al., 1991; Canciu et al., 2022). Enterobactin protects *E. coli* against the oxidative stress induced by various stressors such as H_2_O_2_ or certain agricultural chemicals (Peralta et al., 2022). Iron-free enterobactin can specifically exert cytotoxic effects on highly proliferative cells owe to its significant iron chelating properties (Saha et al., 2019). Thus, enterobactin could be used as a potent anti-cancer agent.

The skeleton of enterobactin is biosynthesized by the extensively studied nonribosomal peptide synthetase (NRPS)-dependent pathway (Kadi and Challis, 2009). Enterobactin synthetase is a two-module NRPS responsible for siderophore biosynthesis in *E. coli* (Crosa and Walsh, 2002). The NRPS components EntB, EntE, and EntF are induced for enterobactin synthesis under iron deficient conditions. At their physiological concentrations, a large amount of linear by-products were produced *in vitro*, including the DHB-Ser trimer, monomer, and dimer (Guo et al., 2008). Since enterobactin is considered a persistence or colonization factor for phytobacterial pathogens such as *Salmonella*, the enterobactin biosynthetic pathway has been suggested as a target for antimicrobial intervention (Quadri, 2007; Hao et al., 2012). Enterobactin also serves as a precursor for the synthesis of the post-translationally modified siderophores such as microcin E492 and salmochelin S4 via glycosylation, linearization or hydrolysis. The resulting siderophores have evolved to evade the host immune response or contribute to host virulence (Delorenzo and Pugsley, 1985; Vassiliadis et al., 2007).

*Streptomyces* are a group of filamentous Gram-positive bacteria with the potential to produce bioactive natural products, including siderophores (Barka et al., 2016). However, the metabolic potential has been underestimated because many metabolites are poorly expressed under laboratory culture conditions. Here, we fermented *S. varspviensis* in different media and found that one of the media triggered the production of catechol siderophores. Siderophores are normally synthesized by microorganisms in response to iron deficiency, and are excreted into the extracellular environment to chelate iron (Sheikh and Taylor, 2009). Studies of bacterial siderophore production are typically performed using iron-limited or iron-deficient media, followed by detection of siderophore production. In our work, five catechol siderophores were purified and their structures were elucidated. These siderophores with distinctive modifications including methylation or oxidation not only enrich the existing diversity of enterobactin siderophore families, but also account for the variation in iron chelating activity. Furthermore, compound **2**, one of the linear enterobactin congeners, exhibited moderate activities against *Listeria monocytogenes*, which are associated with foodborne outbreaks.

## 2. Materials and methods

### 2.1. General experimental procedures

High performance liquid chromatography (HPLC) was conducted on a Waters HPLC system equipped with using an Agilent Eclipse XDB-C18 column (4.6 × 150 mm, 5 μm) for analysis and Fisher X-bridge C18 column (10 × 250 mm, 5 μm) for semi-preparation. Multi-wavelength monitoring was performed at 210, 254, 280 and 330 nm. High resolution electrospray ionization mass spectrometry (HR-ESI-MS) was performed on an Agilent Q-TOF HPLC mass spectrometer. NMR data were recorded on a Bruker 700 MHz system. The structures of enterobactin and congers were elucidated by a combination of HRMS and 1D and 2D NMR spectroscopic analyses. Size exclusion chromatography was performed on Sephadex LH-20 columns (GE Healthcare). Optical rotations were measured with the P-2000 high-accuracy multi-option digital polarimeter (JASCO, Japan). Gene clusters prediction from *S. varsoviensis* CGMCC 4.1431 (Accession no. ASM71863v1) was performed using antiSMASH (Blin et al., 2019). Sequence alignments of the protein sequences deduced from gene clusters were manually annotated by BLAST search (Altschul et al., 1997).

### 2.2. Bacterial strains, and culture conditions

The *S. varsoviensis* CGMCC 4.1431 strain was obtained from the China General Microbiological Culture Collection Center. The strain was grown on medium B (yeast extract 2 g/L, tryptone 2 g/L, glucose 20 g/L, MgSO_4_·7H_2_O 0.5 g/L, K_2_HPO_4_·3H_2_O 1 g/L, pH 7.2) at 30°C for 7 days for sporulation. The spores from one agar plate were harvested, and suspended in 1 mL 20% glycerol. 20 μl of spore suspension was transferred to 50 mL TSB (trypic soy broth) media at 30°C for 2 days as seed culture. Medium B and other two media (Media A: yeast extract 5 g/L, tryptone 5 g/L, glucose 10 g/L, pH 7.0; Media C: soybean meal 10 g/L, sucrose 20 g/L, KCl 8 g/l, pH 7.0) were used for fermentation and comparison (Supplementary Figure S1).

### 2.3. Fermentation, HPLC analysis, and isolation

The spores were harvested and the seed cultures were obtained by inoculating 10 μL of spore solution into 30 mL of TSB liquid medium and incubated at 30°C for 2 days, after which the resulting seed culture was transferred into production medium at 5% for 7 days fermentation. After the small-scale fermentation (50 mL) of *S. varsoviensis*, the resulting culture broth was centrifuged, and the supernatant was extracted three times with ethyl acetate (EtAC). EtAC was removed by rotary evaporation and the resultant sample was dissolved in methanol and subjected for analysis on a Waters HPLC (10 μL injection). The mobile phase was a binary gradient of acetonitrile (ACN) and 0.1% formic acid (FA) in H_2_O, pumped at 0.6 mL/min through an Eclipse XDB-C18 column (5 μm, 4.6 × 150 mm). Liquid chromatography for HPLC analysis was performed using a 30 min solvent gradient from 10–100% solvent B.

For large-scale fermentation (25 L) of the *S. varsoviensis* strain, the cell mass was harvested by centrifugation and the supernatant was extracted with EtAC. The EtAC crude extract (10 g) was concentrated *in vacuo*, and the residue was applied to a silica gel column and separated using an increasing gradient of petroleum ether: EtAC from 5:1 to 1:5 as the mobile phase. Each 250 mL was collected as a fraction. A total of 17 fractions were collected and each fraction was analyzed by HPLC. Fraction D3 was concentrated (1.3 g) and further purified by a Sephadex LH-20 column using methanol as the mobile phase to give 45 subfractions (6 mL each subfraction) and analyzed by TLC and HPLC with UV detection at 254 nm, 300 nm, and 380 nm. Subfraction 25 and 26 and 41 have been chosen for further purification. Preparative HPLC was performed on a C18 X-bridge column (5 μm, 10 × 250 mm) using a 0.1% formic acid in water/ACN as mobile phase, at a flow rate of 3 ml/min. An increasing elution gradient as follows 0–5 min (40% ACN), 5–6 min (40–45% ACN), 6–11 min, 45% ACN; 11–25 min, 45–100% ACN; 25–27 min, 100% ACN; 27–30 min, 100–40% ACN; 30–35 min, 40% ACN was used to afford compound **1** (5.0 mg), compound **3** (2.0 mg) with retention time at 19.6 min and 15.5 min. Compound **2** (4.9 mg), compound **4** (15.0 mg), compound **5** (23.1 mg) were purified from subfraction 41 with retention time at 20.0 min, 19.0 min and 20.7 min, respectively.

Compound **1**: yellow oil; ${\left[\alpha \right]}_{D}^{23}$=17.62 (*c* = 0.42, MeOH). ^1^H NMR and ^13^C NMR data, see Table 1; HR-ESI-MS [M + H]^+^ ion at *m*/*z* 479.1299 (calculated for C_21_H_23_N_2_O_11_^+^, 479.1296).

**Table 1:** The ^1^H NMR (700 MHz) and ^13^C NMR data of 1 and 2.

No.	1*^a^*		2*^a^*	
	*δ*_C_, type	*δ*_H_ (multi, *J* in Hz)	*δ*_C_, type	*δ*_H_ (multi, *J* in Hz)
1	171.0, C		170.9, C	
2	117.2, C		117.1, C	
3	149.9, C		149.9, C	
4	147.3, C		147.3, C	
5	120.0, CH	6.96 (d, 7.8)	120.0, CH	6.95 (*m*)^b^
6	119.9, CH	6.74 (t, 8.0)	119.9, CH	6.73 (*m*)^b^
7	119.5, CH	7.33 (d, 8.2)	119.7, CH	7.30 (*m*)^b^
8	171.1, C		171.0, C	
9	53.3, CH	5.05 (dd, 5.3, 3.9)	53.2, CH	5.00 (t, 4.9)
10	65.1, CH_2_	4.57 (dd, 11.4, 5.3)	65.4, CH_2_	4.53 (dd, 11.4, 5.9)
		4.78 (dd, 11.4, 3.9)		4.74 (m)^b^
11	53.2, CH_3_	3.73 (s)	53.2, CH_3_	3.73 (s)
1’	170.8, C		170.3, C	
2’	116.7, C		116.6, C	
3’	149.6, C		149.5, C	
4’	147.2, C		147.2, C	
5’	120.0, CH	6.96 (d, 7.8)	119.9, CH	6.95 (*m*)^b^
6’	119.9, CH	6.74 (t, 8.0)	119.9, CH	6.73 (*m*)^b^
7’	119.5, CH	7.33 (d, 8.2)	119.6, CH	7.30 (*m*)^b^
8’	171.5, C		171.6, C	
9’	56.6, CH	4.75 (t, 4.5)	56.5, CH	4.72 (t, 4.3)
10’	62.7, CH_2_	3.95 (dd, 11.3, 4.0)	62.7, CH_2_	3.91 (*m*)
		4.04 (dd, 11.4, 4.9)		4.00 (*m*)
1”			170.7, C	
2”			116.8, C	
3”			149.7, C	
4”			147.2, C	
5”			120.0, CH	6.95 (*m*)^b^
6”			119.9, CH	6.73 (*m*)^b^
7”			119.9, CH	7.30 (*m*)^b^
8”			171.1, C	
9”			53.4, CH	5.03 (t, 4.7)
10”			65.0,CH_2_	4.61 (dd, 11.5, 5.5)
				4.76 (*m*)^b^

a Data measured at 700 MHz (^1^H) and 175 MHz (^13^C) in CD_3_OD.

b Overlap.

Compound **2**: Yellow oil; ${\left[\alpha \right]}_{D}^{23}$=12.49 (*c* = 0.41, MeOH). ^1^H NMR and ^13^C NMR data, see Table 1; HR-ESI-MS [M + H]^+^ ion at *m*/*z* 702.1775 (calculated for C_31_H_32_N_3_O_16_^+^, 702.1777).

Compound **3**: ^1^H NMR (700 MHz, CD_3_OD): *δ*_H_ 7.36 (d, *J* = 8.1 Hz, 1H), 6.97 (d, *J* = 7.9 Hz, 1H), 6.77 (t, *J* = 8.0 Hz, 1H), 4.76 (t, *J* = 4.3 Hz, 1H), 4.03 (dd, *J* = 11.4, 4.7 Hz, 1H), 3.95 (dd, *J* = 11.4, 3.9 Hz, 1H), 3.79 (s, 3H).^13^C NMR (175 MHz, CD_3_OD): *δ*_C_ 172.4, 170.6, 149.6, 147.3, 119.8, 119.8, 119.8, 117.2, 62.8, 56.5, 52.9. HR-ESI-MS [M + H]^+^ ion at *m/z* 256.0818 (calculated for C_11_H_14_NO_6_^+^, 256.0816).

Compound **4**: ^1^H NMR (700 MHz, CD_3_OD): *δ*_H_ 7.38 (d, *J* = 8.1 Hz, 1H), 7.33 (d, *J* = 8.1 Hz, 1H), 6.95 (m, 2H), 6.74 (dt, *J* = 16.2, 8.0 Hz, 2H), 6.56 (s, 1H), 5.97 (s, 1H), 5.05 (dd, *J* = 6.0, 3.9 Hz, 1H), 4.79 (dd, *J* = 11.3, 3.9 Hz, 1H), 4.67 (dd, *J* = 11.2, 5.9 Hz, 1H).^13^C NMR (175 MHz, CD_3_OD): *δ*_C_ 172.1, 170.7, 167.9, 164.9, 149.5, 147.8, 147.2, 147.1, 133.4, 121.1, 120.3, 120.3, 119.9, 119.9, 119.8, 119.0, 117.1, 111.3, 66.0, 53.1. HR-ESI-MS [M + H]^+^ 447.1038 (calculated for a molecular formula of C_20_H_19_N_2_O_10_^+^, 447.1034).

Compound **5**: ^1^H NMR (700 MHz, CD_3_OD): *δ*_H_ 7.25 (d, *J* = 8.2 Hz, 3H), 6.97 (d, *J* = 7.8 Hz, 3H), 6.73 (t, *J* = 7.9 Hz, 3H), 5.04 (t, *J* = 5.0 Hz, 3H), 4.66 (d, *J* = 5.0 Hz, 6H). ^13^C NMR (175 MHz, CD_3_OD): *δ*_C_ 170.7, 170.7, 149.5, 147.3, 120.1, 120.1, 119.7, 116.8, 65.8, 53.7. HR-ESI-MS [M + H]^+^ 670.1516 (calculated for C_30_H_28_N_3_O_15_^+^, 670.1515).

### 2.4. Peptide hydrolysis and analysis of Marfey’s assay

The absolute configuration of compounds **1** and **2** was determined by using the Marfey’s assay with minor modifications (Bhushan and Bruckner, 2004). Compounds **1** and **2** were hydrolyzed by heating with HCl (6 N, 1 mL) at 80°C overnight and then the hydrolysis solution was evaporated to dryness, respectively. The resulting residue was redissolved in 100 μl NaHCO_3_ solution (1 M), followed by the addition of 50 μL FDAA solution (1-fuoro-2,4-dinitrophenyl-5-L-alanine amide, 0.2% solution in acetone), and incubated at 55°C for 1 h. The derivatized solution was quenched by the addition of 100 μl HCl (2 M). Amino acid standards of L-Ser and D-Ser were derivatized in the same procedure. A 15 μL aliquot of such reaction mixture was subjected to HPLC analysis (340 nm). HPLC analyses were performed on an Agilent Eclipse XDB-C18 column (5 μm, 4.6 × 150 mm) at a flow rate of 0.6 mL/min. Solvent A consisted of 0.1% formic acid with water, and solvent B was ACN. For the derivatized hydrolysate and the corresponding standards, the following gradient was used for HPLC analysis: 0–5 min, 20% B; 5–35 min, 20–55% B; 35–36 min, 55–100% B; 36–39 min, 100% B; 39–40 min, 100–20% B; 40–45 min, 20% B.

### 2.5. Biological assays of catecholate siderophores from *Streptomyces varsoviensis*

The antibacterial activities were determined in the 96-well plate with LB broth. Each compound for testing was prepared to a concentration of 1 mg/mL in methanol. Five Gram-negative bacteria (*Salmonella enterica* ATCC 14028, *Shigella dysenteriae* CMCC 51335, *Klebsiella pneumonia* HS11286, *Acinetobacter baumannii* ATCC 19606, *Pseudomonas aeruginosa* PAO1) and three Gram-positive bacteria (*Enterococcus faecalis* ATCC 51299, *Staphylococcus aureus* ATCC 25923, and *Listeria monocytogenes* AB97021) were set for bioassay. All the tested strains were grown in LB broth to early stationary phase, diluted by LB broth to ~1.5 × 10^5^ CFU/mL, and then inoculated into 96-well plate with 200 μL of LB in the well. The tested compounds with different concentrations in each wells using a serious dilution (100, 50, 25, 12.5, 6.25, 3.125, 1.5625 μg/mL). No compound but methanol was used as negative control. The MIC (minimum inhibitory concentration) values were evaluated after incubation at 37°C for 16–18 h, using a plate reader at OD_600_.

Evaluation of the iron chelating activities of the different enterobactin-derived compounds was performed using the chrome azurol S (CAS) assay as previously described with some modifications (Andrews and Duckworth, 2016; Murakami et al., 2021). The CAS solution (0.12 mM CAS, 0.3 mM NH_4_AC, 0.3 mM 3-(N,N-Dimethyldodecylammonio) propanesulfonate (DDAPS), 20 μM FeCl_3_) was prepared in distilled Milli-Q water. Each compound was diluted to gradient concentration (approximately 0 to 100 μM), mixed with the CAS solution, and then added to a 96 well-microplate for 1 h incubation at room temperature. The chelating activity of each siderophore compound was calculated according to the absorption at OD_630_ nm using the Synergy H1 multimode reader (BioTek).

## 3. Results

### 3.1. *In silico* analysis of biosynthetic gene clusters of siderophores in *Streptomyces varsoviensis*

**Figure 2:**
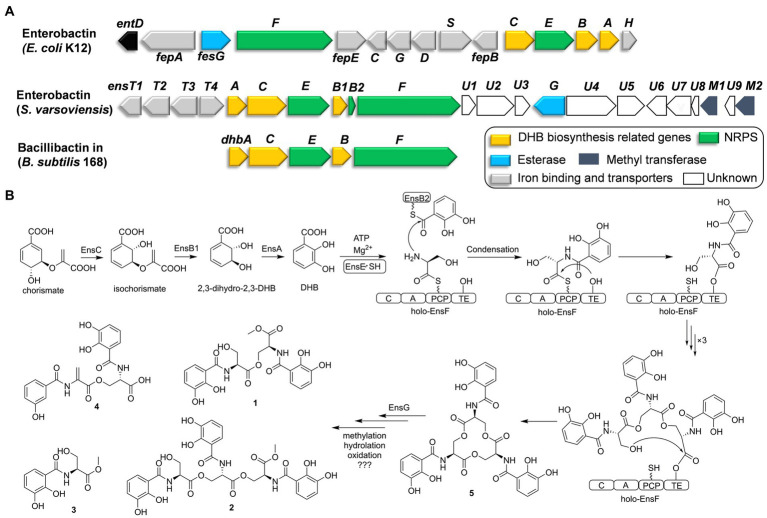
Comparative analysis of catechol siderophore biosynthetic gene clusters to the enterobactin and bacillibactin gene clusters **(A)**. Proposed biosynthetic pathway for the catechol siderophore congers **(B)**.

Genome sequencing of *S. varsoviensis* CGMCC 4.1431 allowed the identification of an NRPS gene cluster containing homologs of catechol siderophore biosynthetic genes (*entA*-*entF*). *In silico* analysis revealed that the NRPS cluster spans approximately ~26 kb, and functional assignments of individual ORFs were made by comparing of the deduced gene products with proteins of known or predicted function (Figure 2A and Supplementary Table S1).

Six genes (*ensA*, *B1*, *B2*, *C*, *E*, *F*) are transcribed in the same orientation and may constitute an operon. Comparative analysis revealed that these genes are significantly identical to their counterparts in the enterobactin or bacillibactin gene cluster. EnsB1 showed 43% identity to an isochorismatase, EnsC is 40% identical to the isochorismate synthase, and EnsA resembles a 2,3-DHB dehydrogenase similar to EntA (45% identity) from *E. coli*. EnsB1, EnsC and EnsA are proposed to be responsible for the biosynthesis of the 2,3-DHB moiety from chorismic acid. *EnsB2* encodes a carrier protein next to EnsB1. EnsB2 is proposed to function as a peptidyl-carrier protein (PCP) for tethering 2,3-DHB because it reveals similarity to the enterobactin synthetase component B EntB (43% identity). EnsE is a stand-alone adenylation (A) domain which is similar to DhbA involved in the catechol siderophore bacillibactin from *Bacillus subtilis* (67% identity/79% similarity) and EntA involved in enterobactin biosynthesis by *E. coli* (48% identity/65% similarity). EnsE catalyzes the formation of an adenylate from 2,3-DHB and tethers to the phosphopantetheine thiol of EnsB2. EnsF consists of a terminate NRPS module (C-A-PCP-TE) and is most similar to the NRPS of DhbF, which is involved in the biosynthesis of *B. subtilis* bacillibactin (46% identity/60% similarity). The featured 10 conserved residues in the amino acid binding pocket predict serine incorporation by EnsF. A TE domain at the C-terminal end of EnsF is likely responsible for the macrocyclization. EnsG is an esterase similar to enterobactin esterase that catalyzes the hydrolysis to convert cyclic enterobactin to linear form in *E. coli* (Kadi and Challis, 2009; Reitz et al., 2017).

EnsT1-T4 could be formed the transport system homologous to FepBCDG for the ferri-siderophore complex (Krewulak and Vogel, 2008). EnsT1 is a Fe^3+^-citrate import ATP-binding protein. EnsT2 and T3 are permease proteins of the siderophore transport system. EnsT4 is a siderophore-binding protein. Additionally, *ensU1* to *U9*, and two methyltransferases encoded by *ensM1* and *M2* are located in the downstream of these core genes. These genes appear to be present within the cluster in *S. varsoviensis*, but their functions remain unknown (Figure 2A).

### 3.2. Isolation and structural elucidation of enterobactin derivatives from *Streptomyces varsoviensis*

In our search for the metabolic potential of *S. varsoviensis*, the varying metabolic profiles in three different culture media were compared by HPLC analysis (Supplementary Figure S1). Of these, medium A showed a novel chemical profile that was not present in the other two media, further guiding the medium selection for large-scale fermentation, and ultimately yielding five of the catechol siderophore congeners, including two new compounds (**1** and **2**, Figure 1).

Compound **1** was obtained as yellow oil. HR-ESI-MS analysis of **1** in positive mode gave an [M + H]^+^ ion at *m*/*z* 479.1299 (calculated 479.1296 for C_21_H_23_N_2_O_11_^+^), requiring 12 degrees of unsaturation. The molecular formula was established as C_21_H_22_N_2_O_11_. The UV spectrum displayed absorptions at λ_max_ 207.5, 247.7, and 311.9 nm similar to catecholate metabolites (O’Brien and Gibson, 1970). The ^1^H NMR spectrum showed one methyl proton in the upfield (*δ*_H_ 3.73, *s*), and the existence of two aromatic rings was supported by three aromatic protons in the downfield *δ*_H_ 7.33 (d, *J* = 8.2 Hz), 6.96 (d, *J* = 7.8 Hz) and 6.74 (t, *J* = 8.0, 1.8 Hz) with equal integrated area and. Analysis of the ^13^C NMR spectrum indicated the existence of 21 carbons attributable to four carbonyl carbons and 12 olefinic bond signals. Together with the DEPT135 and HSQC spectra, all the carbons were identified as one methyl (*δ*_C_ 53.2), two methylenes (*δ*_C_ 65.1 and 62.7), eight methines (*δ*_C_ 120.0, 120.0, 119.9, 119.9, 119.5, 119.5, 56.6, and 53.3) and 10 quaternary carbons (*δ*_C_ 171.5, 171.1, 171.0, 170.8, 149.9, 149.6, 147.3, 147.2, 117.2, and 116.7). The planar structure of **1** was assigned after careful analysis of the 2D NMR (HSQC, ^1^H–^1^H COSY, HMBC, ROESY) spectra. Four spin coupling systems were confirmed by the ^1^H-^1^H COSY cross-peaks between H-5/H-6/H-7, H-9/H-10, H-5’/H-6’/H-7′, and H-9’/H-10′ (Supplementary Figures S2–S8, and S23). The HMBC revealed the correlations of H5 with C3, H6 with C2 and C4, H7 with C1 and C3, H9 with C1, H10 with C8 and C8’, H-11 and C-8, H-5′ with C-3′, H-6′ with C-2′ and C-4′, H-7′ with C-1′ and C-3′, H9’ with C8’, H-10′ with C-8′. Taken together, two 2,3-dihycroxybenzoyl substructure and a serine residue were established in **1**. HMBC correlations from the α-CH proton (*δ* 4.75) to the C9’ and α-CH proton (*δ* 5.05) to the C9 carboxyl suggested that an amide linkage connected the three fragments. All the four spin-coupling fragments were connected together, and **1** was finally established as a new enterobactin derivative (Table 1 and Supplementary Figures S2–S8).

The absolute configuration of **1** was determined by Marfey’s analysis. After complete acid hydrolysis of compound **1**, the released amino acids were derivatized with Marfey’s reagent (L-FDAA) and compared with L-and D-Ser amino acid standards under the same conditions. Comparison of the retention time of the **1** hydrolysate and amino acid standards using HPLC analysis revealed an L-configuration for the serine residue in **1**. The absolute configuration at C9 of **1** was determined as 9*S*, 9’*S*. Thus, **1** was named as N,N′-bis(*S*-2,3-dihydroxybenzoyl)-*O*-seryl methoxy serinate.

Compound **2** was obtained as yellow oil. It had a molecular formula of C_31_H_31_N_3_O_16_ based on the ion peak at *m/z* 702.1775 (calculated *m/z* 702.1777 for [M + H]^+^), indicating 18 degrees of unsaturation. The UV spectrum displayed absorption bands at λ_max_ 210, 250, and 314 nm, suggesting the presence of the hydroxylated benzoyl chromophores similar to **1**. The ^1^H NMR spectrum revealed one methyl proton in the upfield (*δ*_H_ 3.73, *s*). The three aromatic rings were readily assigned based on the proton-splitting pattern in *δ*_H_ 7.30 (m), 6.95 (m), and 6.73 (m). NMR data of **2** showed 31 carbons contributing to four carbonyl carbons and 18 olefinic bond signals. These data were very similar to the planar structure of compound **1**. In the ^1^H-^1^H COSY spectrum, six fragments including H-5’/H-6’/H-7′, H-9’/H-10′, H-5”/H-6”/H-7′, H-9”/H-10′, H-9/H-10, and H-5/H-6/H-7, were detected (Supplementary Figures S9–S15, S23). Three typical ^3^*J*_HH_ coupling patterns represent a 1,2,3-trisubstituted benzene ring. Together with the HMBC correlations between H-5 with C-3 and C-4, H-6 with C-2 and C-4, H-7 with C-1 and C-3, H-9 with C-1, H-10 with C-8 and C-8′, H-11 with C-8, H-5′ with C-3′ and C-4′, H-6′ with C-2′ with C-4′, H-7′ with C-1′ and C-3′, H-10′ with C-8′, H-5′ with C-3′ and C-4′, H-6′ with C-2′ and C-4′, H-7′ with C-1′ and C-3′, H-9′ with C-1′, and H-10′ with C-8 and C-8′, **2** was identified as a derivative of **1** with an additional 2,3-dihydroxybenzoyl-L-serine connected to the C9.” All the three fragments were connected together to form the planar structure of **2** by ester bonds based on HMBC correction from H-10 to C-8′ and H-10′ to C-8′. Finally, the absolute configuration of the serine residue in **2** was determined as L-configuration similarly using Marfey’s analysis. The absolute configuration at C9 of **2** was determined as 9*S*, 9’*S*, and 9”*S*, respectively. Thus, **2** was designated as N,N′,N′-tris(*S*-2,3-dihydroxybenzoyl)-*O*-seryl-*O*-seryl methoxy serinate.

Compound **3** was obtained as yellow and amorphous solid. HR-ESI-MS analysis of **3** afforded an [M + H]^+^ ion at *m/z* 256.0818 (calculated [M + H]^+^ ion at *m/z* 256.0816), establishing its molecular composition as C_11_H_13_NO_6_. According to the ^1^H and ^13^C NMR data (Supplementary Figures S16, S17), compound **3** had the same 2,3-dihydroxybenzoate moiety as **1** and **2**. By comparison with literature data, compound **3** was identified as methyl-2,3-dihydroxybenzoylserine (Fischbach et al., 2005). Compound **4** was isolated as a yellow and amorphous solid. HR-ESI-MS analysis yielded an [M + H]^+^ ion at *m/z* 447.1038, consistent with a molecular formula of C_20_H_18_N_2_O_10_ (calculated [M + H]^+^ ion at *m/z* 447.1034). Compound **4** was identified as N,N′-bis(2,3-dihydroxybenzoyl)-*O*-L-seryl-L-dehydroalanine, the derivative of enterobactin, based on the comparison of the NMR and MS data with previously published data (Asamizu et al., 2022). Compound **5** was obtained as yellow and amorphous solid. HR-ESI-MS analysis of compound **5** afforded an [M + H]^+^ ion at 670.1516 (calculated [M + H]^+^ ion at *m*/*z* 670.1515), giving a molecular formula of C_30_H_27_N_3_O_15_. Based on the comparison of its NMR and MS data with previously published data, compound **5** was identified as known compound enterobactin (Bergstrom et al., 1991).

### 3.3. Bioactivity evaluation of catecholate-type siderophores

For evaluation of antimicrobial activity, **1** and **2** were tested for antibacterial activity against five Gram-negative bacteria, including *Salmonella enterica* ATCC 14028, *Shigella dysenteriae* CMCC 51335, *Klebsiella pneumonia* HS11286, *Acinetobacter baumannii* ATCC 19606, *Pseudomonas aeruginosa* PAO1, and three Gram-positive bacteria, including *Enterococcus faecalis* ATCC 51299, *Staphylococcus aureus* ATCC 25923, and *Listeria monocytogenes* AB97021, using a serial dilution method in the 96-well plate. **1** showed no activity against all the tested strains (MIC >100 μg/mL) while **2** showed moderate activity against *Listeria monocytogenes* AB97021, with MIC at 25 μg/mL, but not against other tested Gram-negative or Gram-positive strains (MIC >100 μg/mL) (Supplementary Table S2). The MIC of **2** is similar to the reported MIC value for sulfisoxazole (Vela et al., 2001).

The modified CAS microplate assay was used to access the siderophore activity of compound **1**–**5**. In the assay solution, the siderophore compounds seize the iron from the Fe-CAS-DDAPS complex to form the siderophore-CAS-DDAPS complex, causing the absorbance of CAS solution to decrease at 630 nm. The colorimetric shift is correlated with the iron chelating activity of each compound. Thus, compounds **1** and **2** showed higher iron chelating activity than enterobactin (Supplementary Figure S24).

## 4. Discussion

In the natural environment, microorganisms constantly secrete small functional molecules to communicate directly or indirectly with each other or to adapt to specific niches. Siderophores are a group of secondary metabolites used for iron acquisition. The secretion of siderophores as high affinity iron chelators is a key feature of most microbial growth. In this work, we have discovered and isolated catecholate siderophores framed on 2,3-dihydroxybenzoyl-L-serine from *S. varsoviensis*.

Siderophores are types of iron chelators which produced by microorganisms in response to iron deficiency. The characteristic scaffolds of siderophores for ferric iron chelation include catecholates, hydroxamates, α-hydroxy acids, and similar bidentate functional groups. It may be advantageous for bacteria to generate multiple chelators with different iron affinities. *Streptomyces* are metabolically diverse microorganisms capable of generating a range of natural products, including siderophores. We surveyed the entire genome sequence of *S. varsoviensis* and observed several siderophore biosynthetic gene clusters of different types. *In silico* analysis revealed an NRPS biosynthetic gene cluster for siderophores containing the 2,3-dihydroxybenzoate moiety.

In order to evaluate the siderophores produced by *S. varsoviensis* under certain conditions, we tested its growth and fermentation conditions with varying carbon and nitrogen sources. The comparison of metabolite profiles in different media identified a fermentation medium that allowed the production of a series of new peaks with the typical UV spectrum of the dihydroxybenzoate moiety (Supplementary Figure S1). All five compounds (**1**–**5**), which share an *N*-2,3-dihydroxybenzoyl-L-serine as a common structural unit, have been isolated and structurally elucidated. Of these compounds, **1**–**4** are 2,3-DHB monomer, dimer, or linear trimer congeners and **5** is enterobactin.

Enterobactin is one of the well-known tri-catecholate siderophores and its metal-binding property has attracted considerable attention. Enterobactin consists of an L-serine trilactone linked by amide bonds to three 2,3-DHB moieties, and further sequence analysis showed that the *S. varsoviensis* NRPS gene cluster *ens* is in accord with the biosynthetic logic. As shown in Figure 2, the genes directing 2,3-DHB biosynthesis were *ensABC* utilizing chorismite as a precursor, which is derived from the shikimate acid synthesis pathway. The synthesized 2,3-DHB is activated via EntE (A domain) and transferred to the EntB2 (PCP domain). The iterative NRPS EntF activates the L-serine and subsequently catalyzes the formation of each ester and amide bond. Finally, the TE domain finally catalyzes the macrocyclization of the trimer and release of the final enterobactin siderophore.

Siderophore biosynthetic gene clusters, such as the well-known desferrioxamine cluster, are widely distributed throughout most *Streptomyces* species (Barona-Gomez et al., 2004). In contrast, the enterobactin-like gene cluster has only been found in certain *Streptomyces* strains (Fiedler et al., 2001). The presence of the enterobactin-like gene cluster had a much more restricted distribution than desferrioxamine, suggesting that the random horizontal gene transfer events only occurred in some species (Soutar and Stavrinides, 2018). The common evolutionary ancestry of entrobactin is strongly suggested by sequence similarity, which implies considerable structural and functional homology. Orthologs of the enterobactin gene cluster are strongly implied from the sequence similarity, indicating conserved structure and function. The reaction sequence for the biosynthesis of enterobactin by the *ens* operon was shared the high similarity with the enterobactin and bacillibactin gene clusters (Figure 2A). Siderophores are the important functional molecules among the *Streptomyces*. The acquisition of the enterobactin-like gene cluster from Enterobactium by horizontal gene transfer could be a strategy used by *Streptomyces* species to adapt to a specific ecological environment. (Morris et al., 2012).

Considering that horizontal gene transfer influences the evolution of biosynthetic gene clusters, subsequent evolutionary pressures and genetic divergence have led to variation in the gene clusters by combining the genes from the *Streptomyces* recipients (Tidjani et al., 2019; Kramer et al., 2020). Downstream of the *ens* cluster in *S. varsoviensis*, a series of genes with hypothetical functions have been identified. According to the isolated methylated compounds **1**–**3**, the emergence of two methyltransferases EnsM1 and EnsM2 could be responsible for the O-methyl modification. Methylation could be a bacterial strategy to produce a siderophore resistant to a specific environment or to prevent its use by competitive bacteria (Reitz et al., 2017). Gene disruption and complementation would reveal the timing and role of *ensM1* and *ensM2* in the modification steps.

Compounds **1**–**4** are monomers, dimers, and linear trimers from *S. varsoviensis*. These compounds could be released during enterobactin biosynthesis if oligomerisation or cyclisation is interrupted, or produced by enzymatic hydrolysis (Abergel et al., 2009; Reitz et al., 2017; Figure 2). It is worth noting that some bacteria do not synthesize the siderophore themselves, but will use it to scavenge iron in a piracy strategy. Macrolactone hydrolysis alters the physical properties of enterobactin, reduces its membrane affinity and may further facilitate the piracy by enhancing iron uptake from the environment (Luo et al., 2006). Enterobactin esterase hydrolyzes the siderophore, releasing iron for use by the cell. One possibility is that EnsG, the esterase from *S. varsoviensis*, is involved in the hydrolysis. These hydrolyzed products of the catechol siderophore are common type siderophores with bioactivity. For example, catechoserine from *Streptomyces* is a catechol-type inhibitor of tumor cell invasion (Igarashi et al., 2012); chrysobactin is a virulence factor produced by plant pathogens (Persmark et al., 1989). Our antimicrobial assay indicated that compound **2**, the linear trimer, had modest activity against *Listeria monocytogenes*.

## 5. Conclusion

Siderophores are potent compounds with agricultural and medicinal applications. Catechol moieties have been shown to be promising siderophore cores that form bioactive conjugates with antibiotics. Five catecholate siderophores with variation in iron chelating activity have been isolated from *S. varsoviensis*. As a siderophore with a broad range of biological activities such as anticancer and antimicrobial, enterobactin can be exploited as a potential agent for future therapy. Thus, our work will not only extend the existing structural diversity of the enterobactin family of compounds, but will also provide new enzymes for enterobactin derivation and expand the toolbox for the synthetic biology research. Our future investigations will uncover genetic knowledge and the precise function of the genes in the gene cluster.

## Data availability statement

The datasets presented in this study can be found in online repositories. The names of the repository/repositories and accession number(s) can be found in the article/Supplementary material.

## Author contributions

ZL, TH, SL, and ZD designed the experiments. ZL and QS conducted the experiments and analyzed the data. TH, ZL, and SL drafted, reviewed and edited the manuscript. All authors contributed to the article and approved the submitted version.

## Funding

This work was financially supported by grants from the National Key Research and Development Program of China (2018YFA0901900), National Natural Science Foundation of China (22207073), the Program of Shanghai Subject Chief Scientist (21XD1401300) and Major Project of Haihe Laboratory of Synthetic Biology (22HHSWSS00001).

## Conflict of interest

The authors declare that the research was conducted in the absence of any commercial or financial relationships that could be construed as a potential conflict of interest.

## Publisher’s note

All claims expressed in this article are solely those of the authors and do not necessarily represent those of their affiliated organizations, or those of the publisher, the editors and the reviewers. Any product that may be evaluated in this article, or claim that may be made by its manufacturer, is not guaranteed or endorsed by the publisher.
