# Supplementary material for: Catechol siderophores framed on 2,3-dihydroxybenzoyl-L-serine from Streptomyces varsoviensis
Source: Front Microbiol. 2023 May 3;14:1182449. doi: 10.3389/fmicb.2023.1182449 (PMC10188961; doi:10.3389/fmicb.2023.1182449)
Supplement: Supplementary file 1 [file Data_Sheet_1.docx]

Supplementary Material

Catechol siderophores framed on 2,3-dihydroxybenzoyl-L-serine from *Streptomyces varsoviensis*

Zhixiang Liu^1,∇^, Tingting Huang^1,3,∇^, Qing Shi, Zixin Deng^1,3^, Shuangjun Lin^1,2,3,*^

^1^State Key Laboratory of Microbial Metabolism, Joint International Research Laboratory on Metabolic & Developmental Sciences, School of Life Sciences & Biotechnology, Shanghai Jiao Tong University, 800 Dongchuan Road, Shanghai, China

^2^Frontiers Science Center for Transformative Molecules, Shanghai Jiao Tong University, 800 Dongchuan Road, Shanghai 200240, China

^3^Haihe Laboratory of Synthetic Biology, Tianjin 300308, China

^∇^ These authors contributed equally to this work.

*** Correspondence:**

Shuangjun Lin

linsj@sjtu.edu.cn

# Supplementary Figures and Tables

**1.1 Supplementary figures**

**Supplementary Figure 1.** Comparative analysis for *Streptomyces varsoviensis* in three different culture media

**Supplementary Figure 2-8.** 1D- and 2D-NMR spectra for compound **1**.

**Supplementary Figure 9-15.** NMR spectra for compound **2**.

**Supplementary Figure 16-17.** NMR spectra for compound **3**.

**Supplementary Figure 18-19.** NMR spectra for compound **4**.

**Supplementary Figure 20-21.** NMR spectra for compound **5**.

**Supplementary Figure 22.** Marfey's analysis of compound **1** and **2**.

**Supplementary Figure 23.** H−^1^H COSY () and key HMBC () correlations of compound **1** and **2**.

**Supplementary Figure 24.** Detection of the siderophore charity of compound **1**-**5** by CAS assay.

**1.2 Supplementary tables**

**Supplementary Table S1**. Annotation of Enterobactin gene cluster (*ens*) from *S. varsoviensis*.

**Supplementary Table S2**. Antimicrobial activity of **1** and **2**.

## Supplementary Figures


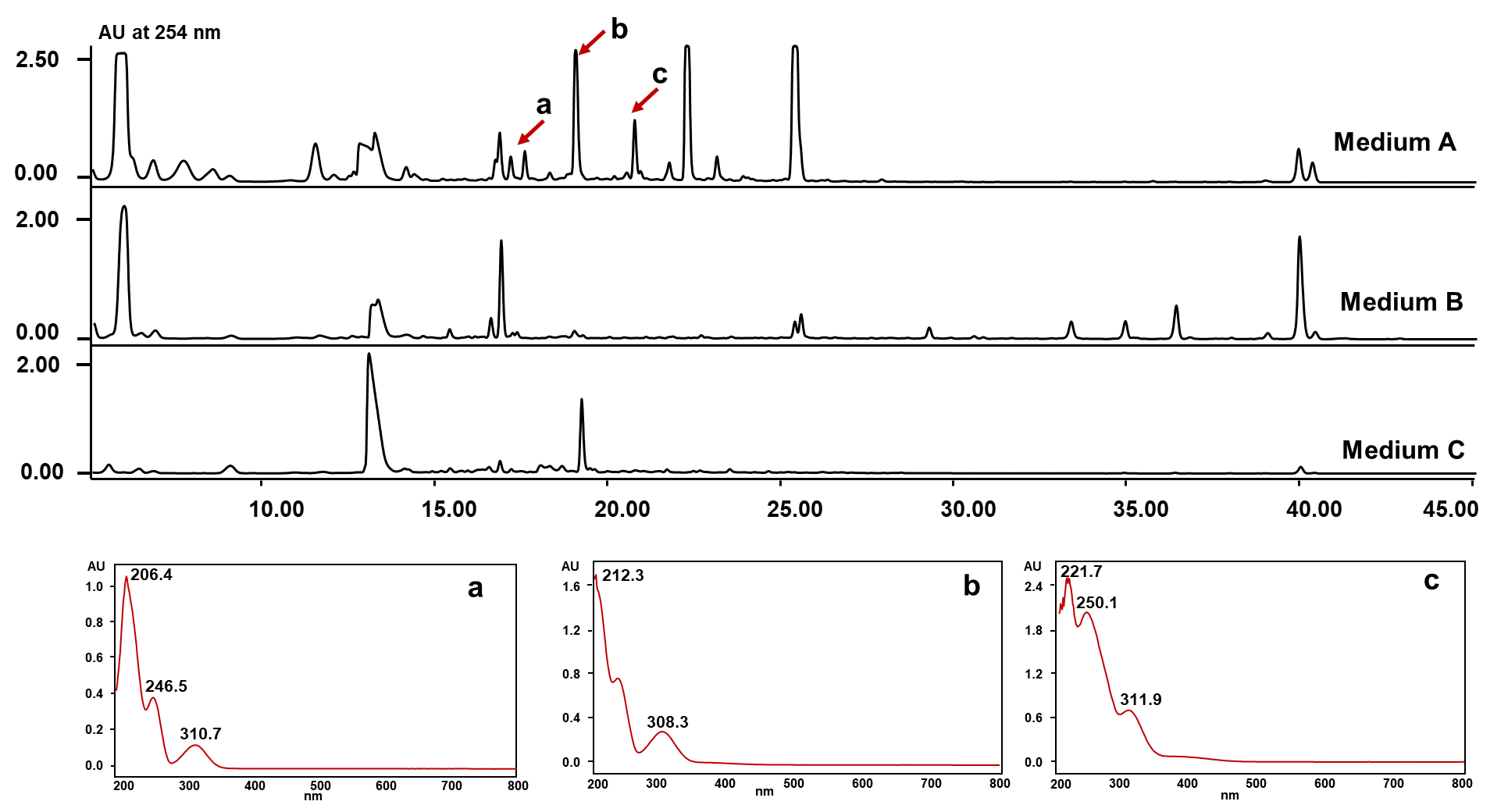


**Supplementary Figure 1. Comparative analysis for *Streptomyces varsoviensis* in three different culture media.** Medium A revealed specific peaks (top). The UV spectrum of peak a, b, and c are listed (bottom).


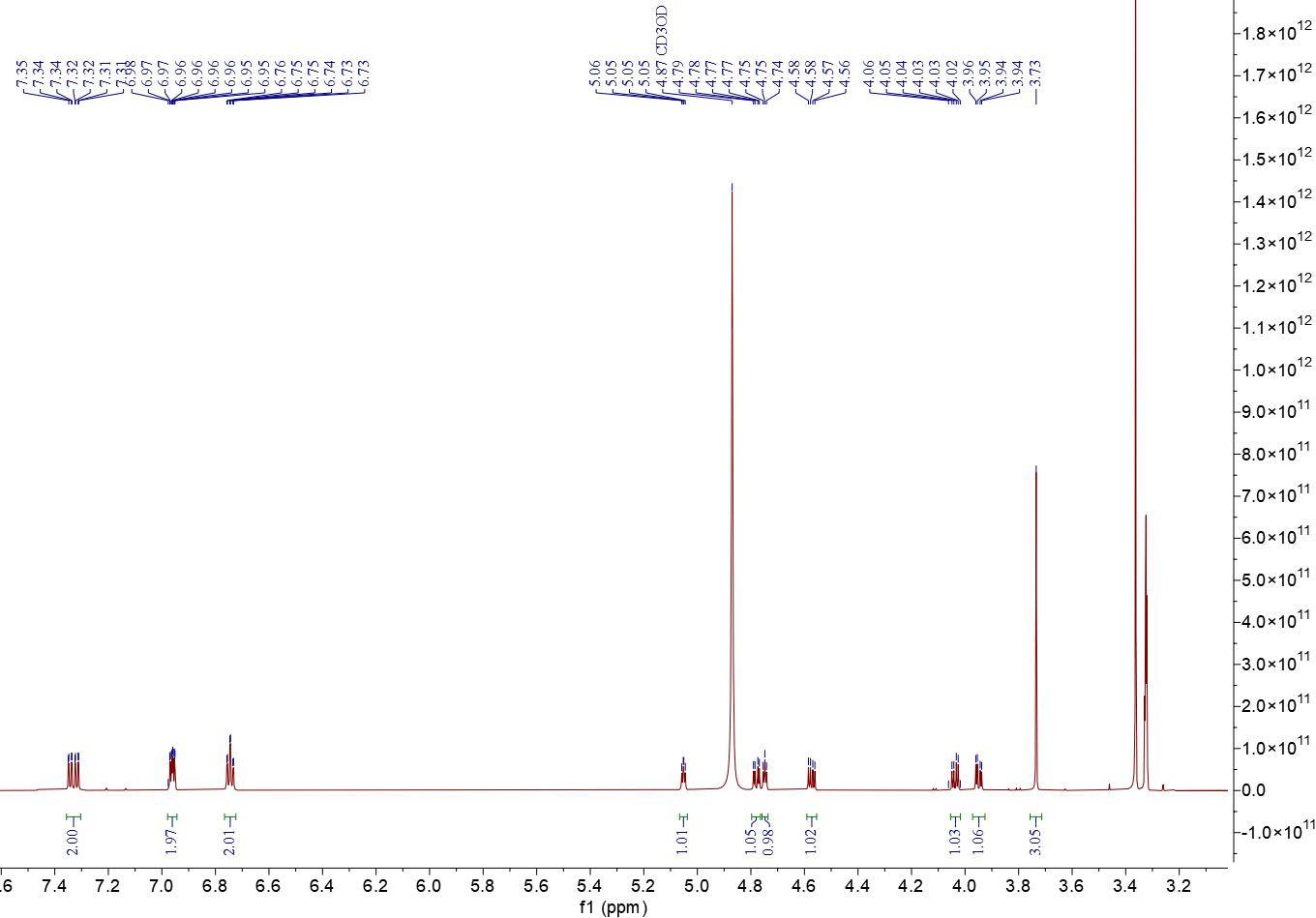


**Supplementary Figure 2.** ^1^H NMR spectrum of compound **1** (700 MHz, CD_3_OD).


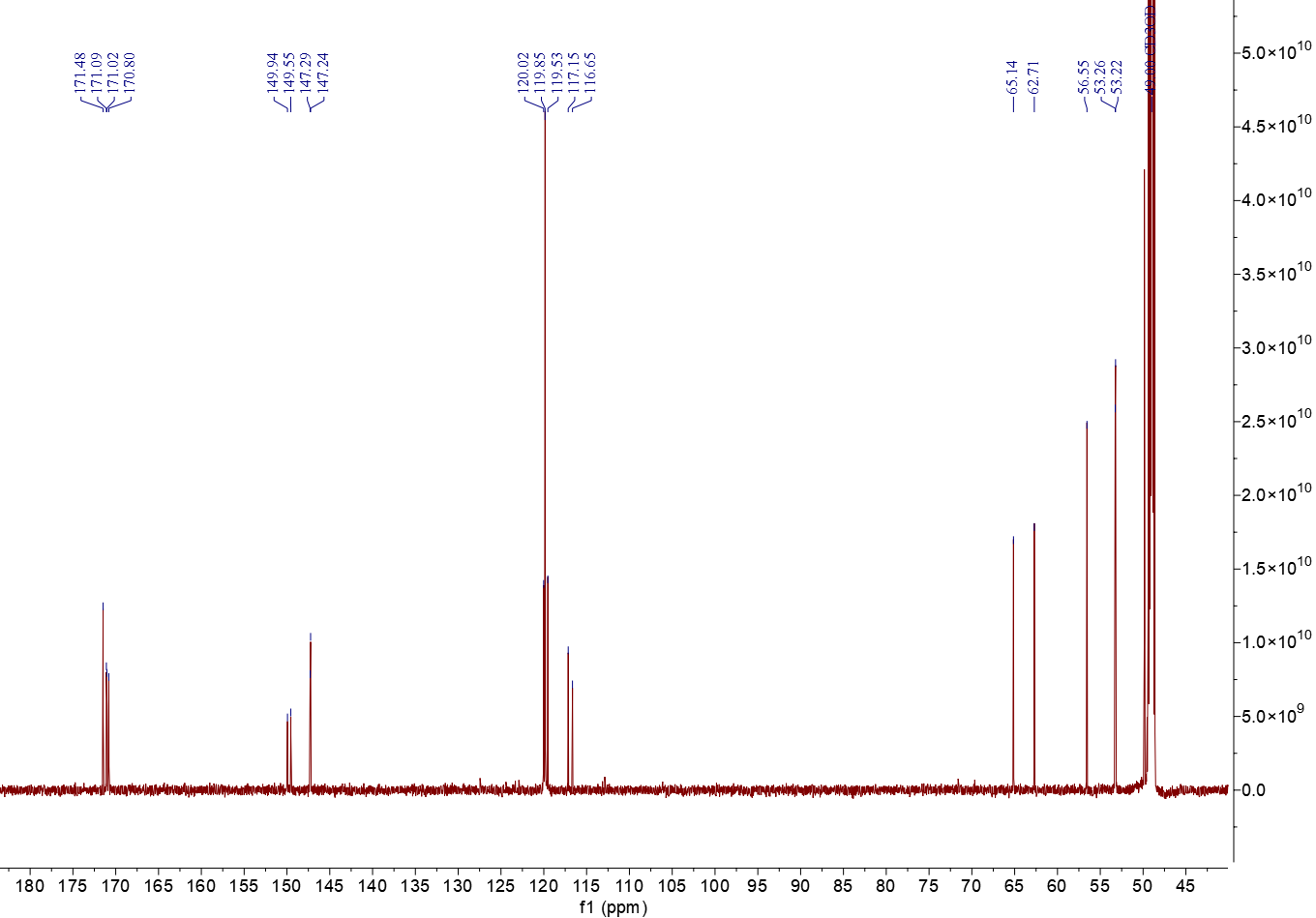


**Supplementary Figure 3.** ^13^C-NMR spectrum of compound **1** (175 MHz, CD_3_OD).


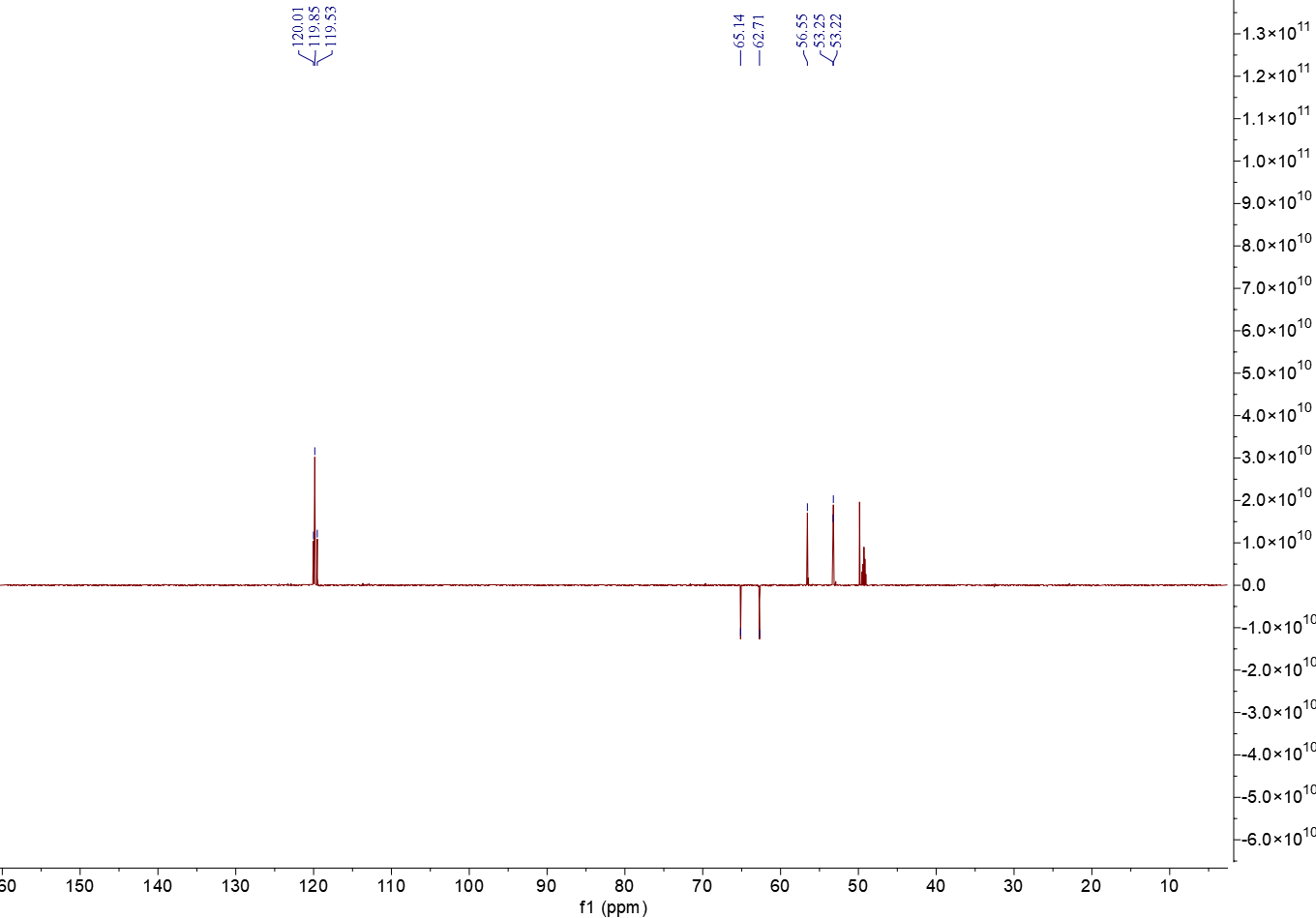


**Supplementary Figure 4.** DEPT135 spectrum of compound **1**.


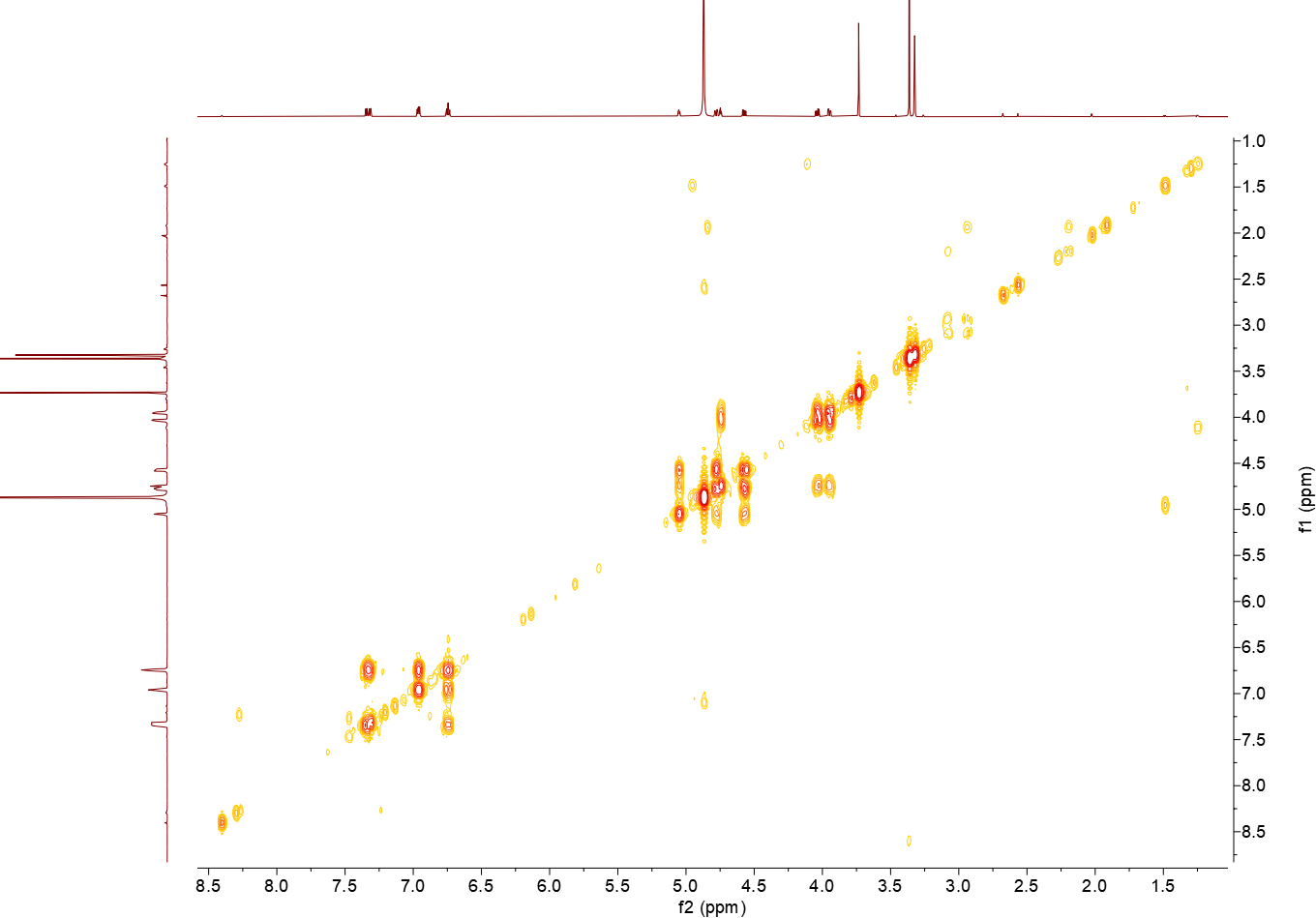


**Supplementary Figure 5.** ^1^H-^1^H COSY spectrum of compound **1**.


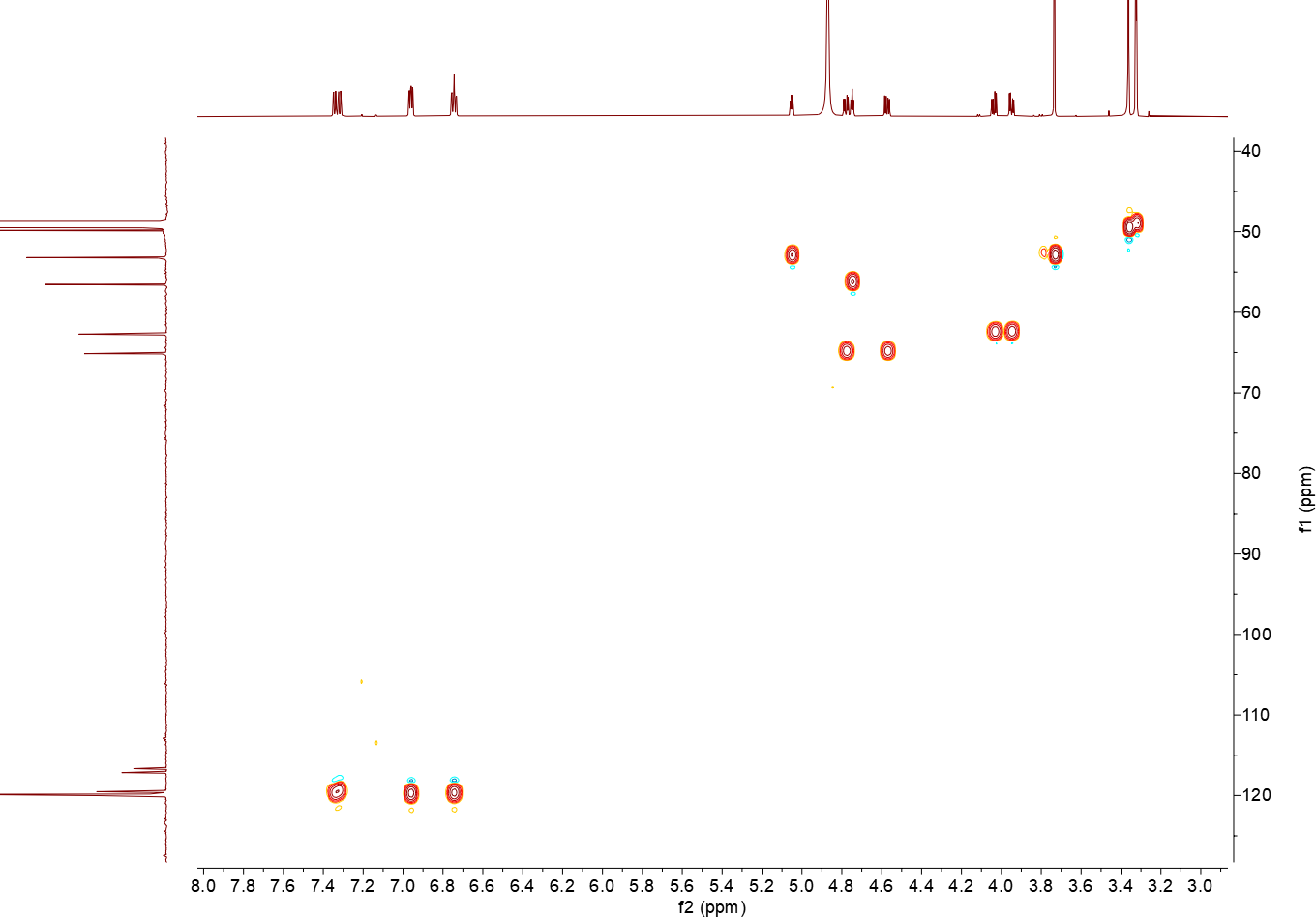


**Supplementary Figure 6.** ^1^H–^13^C HSQC spectrum of compound **1**.


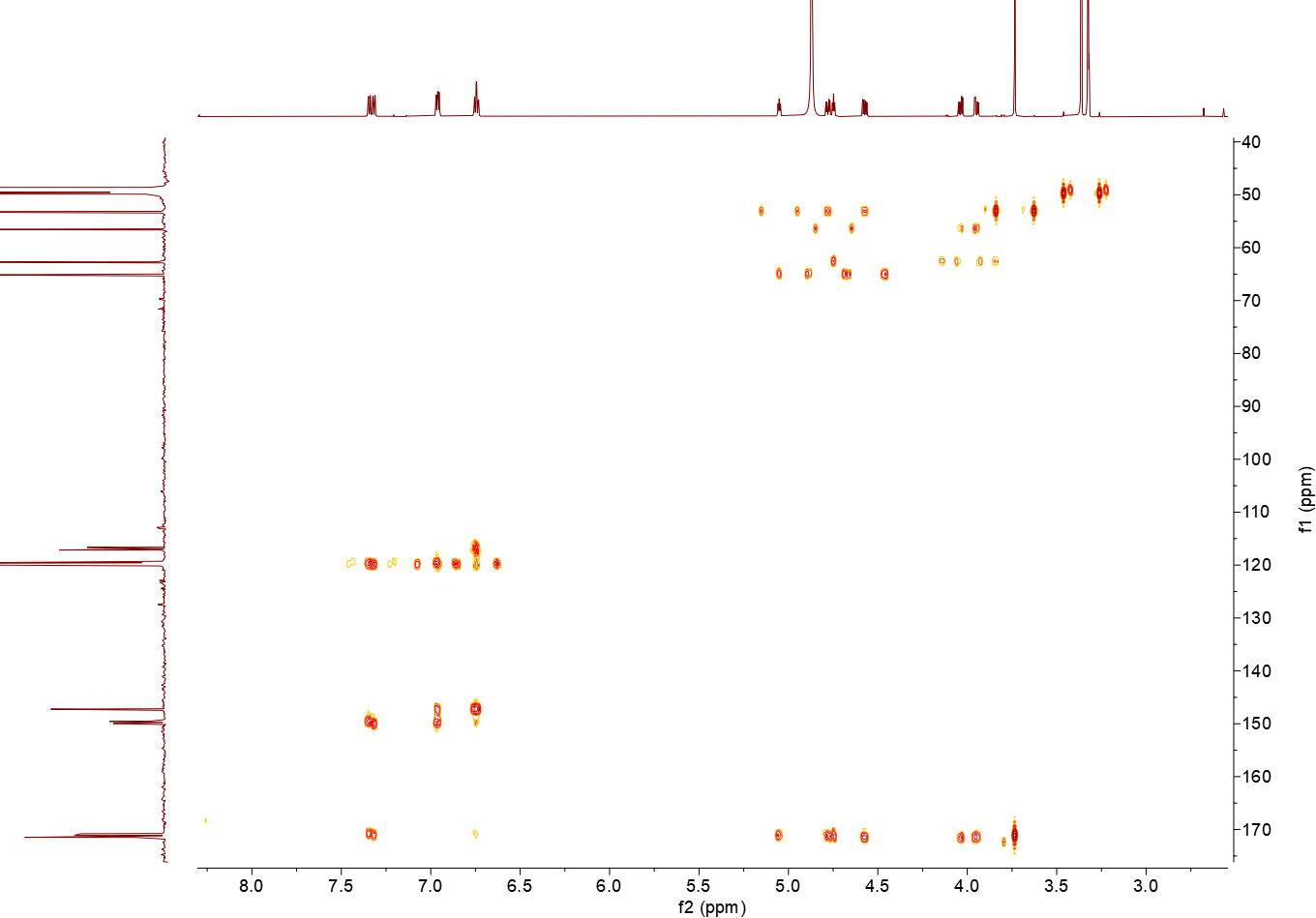


**Supplementary Figure 7.** ^1^H–^13^C HMBC spectrum of compound **1**.


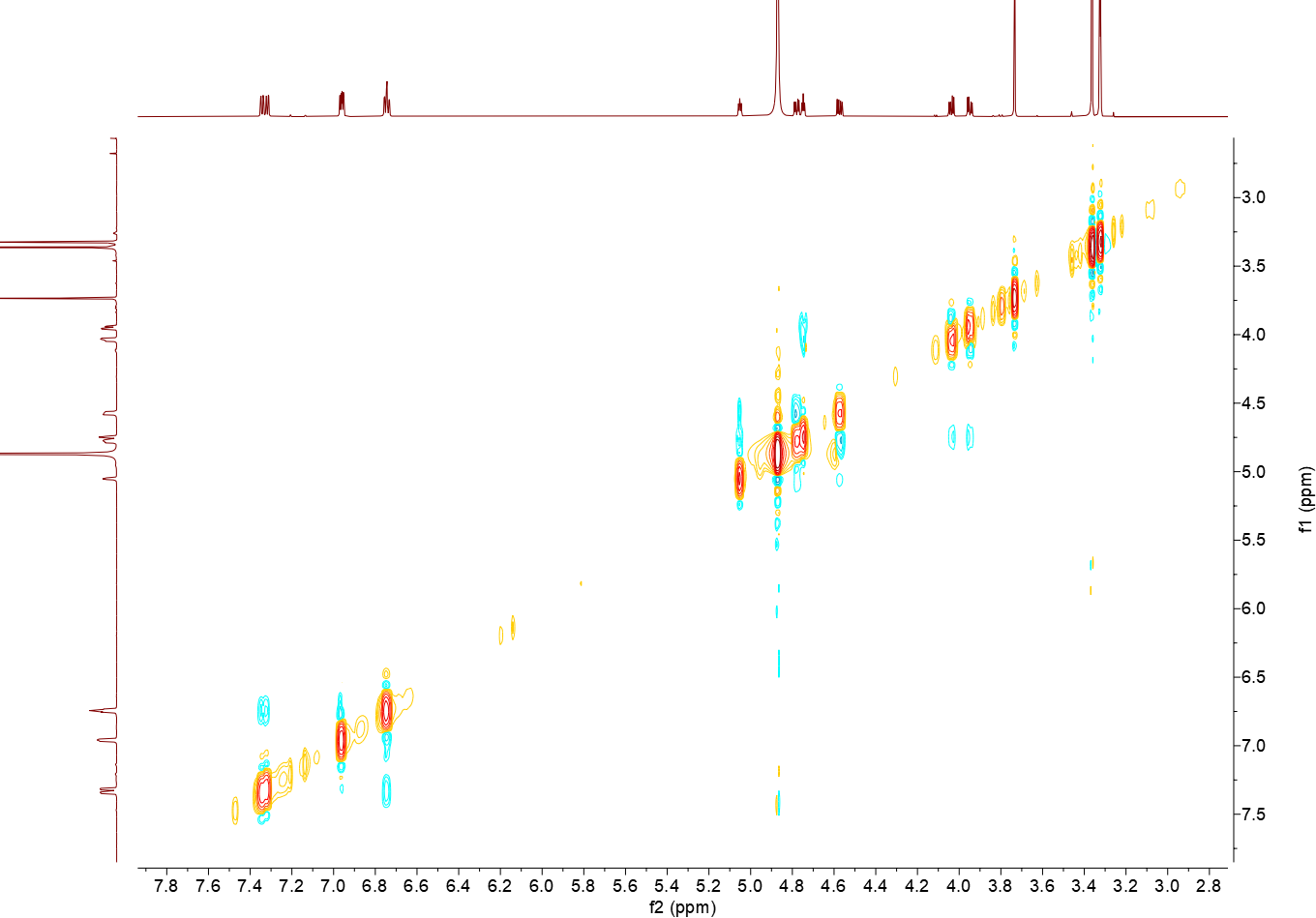


**Supplementary Figure 8.** NOESY spectrum of compound **1**.


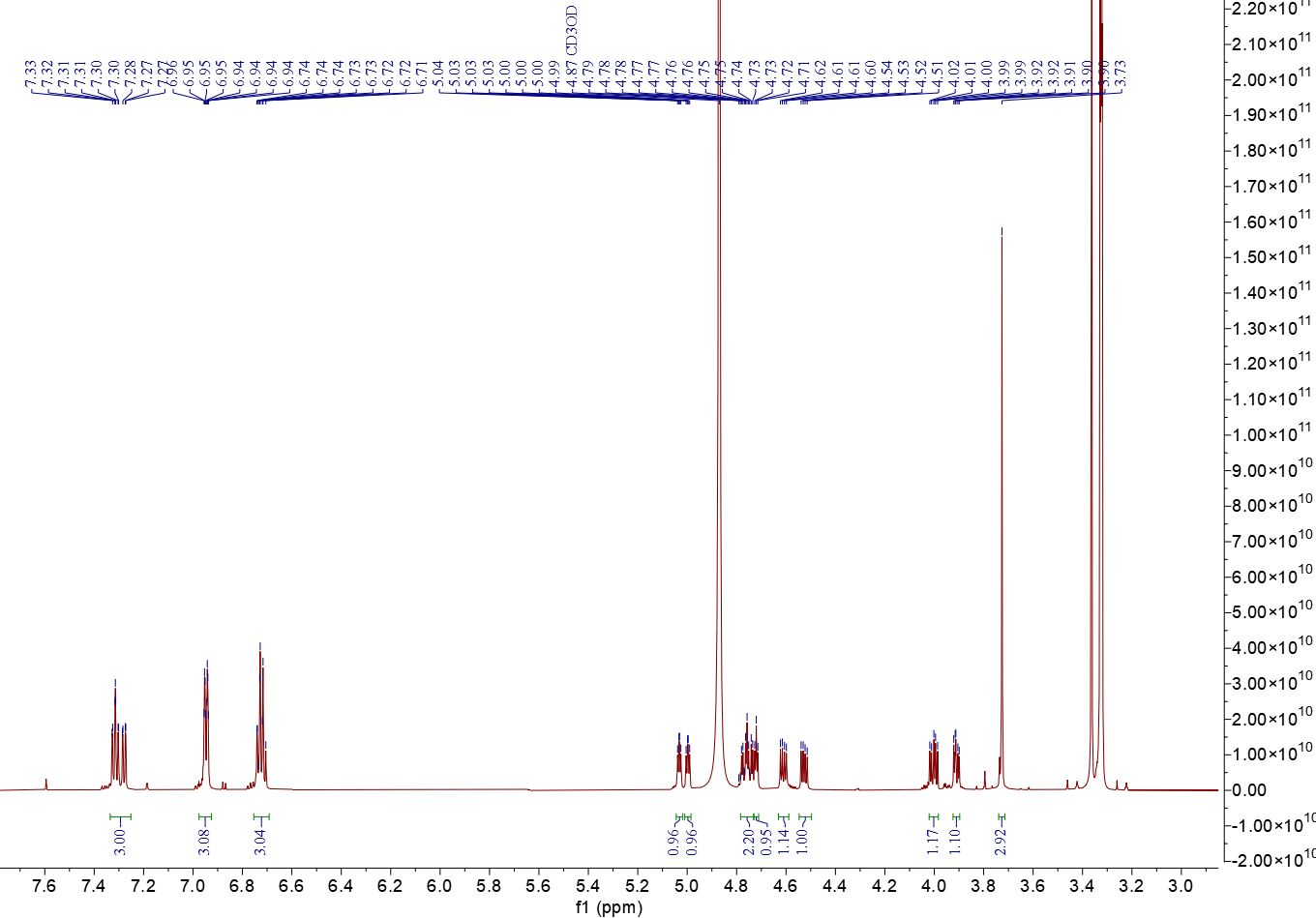


**Supplementary Figure 9.** ^1^H NMR spectrum of compound **2** (CD_3_OD, 700 MHz)


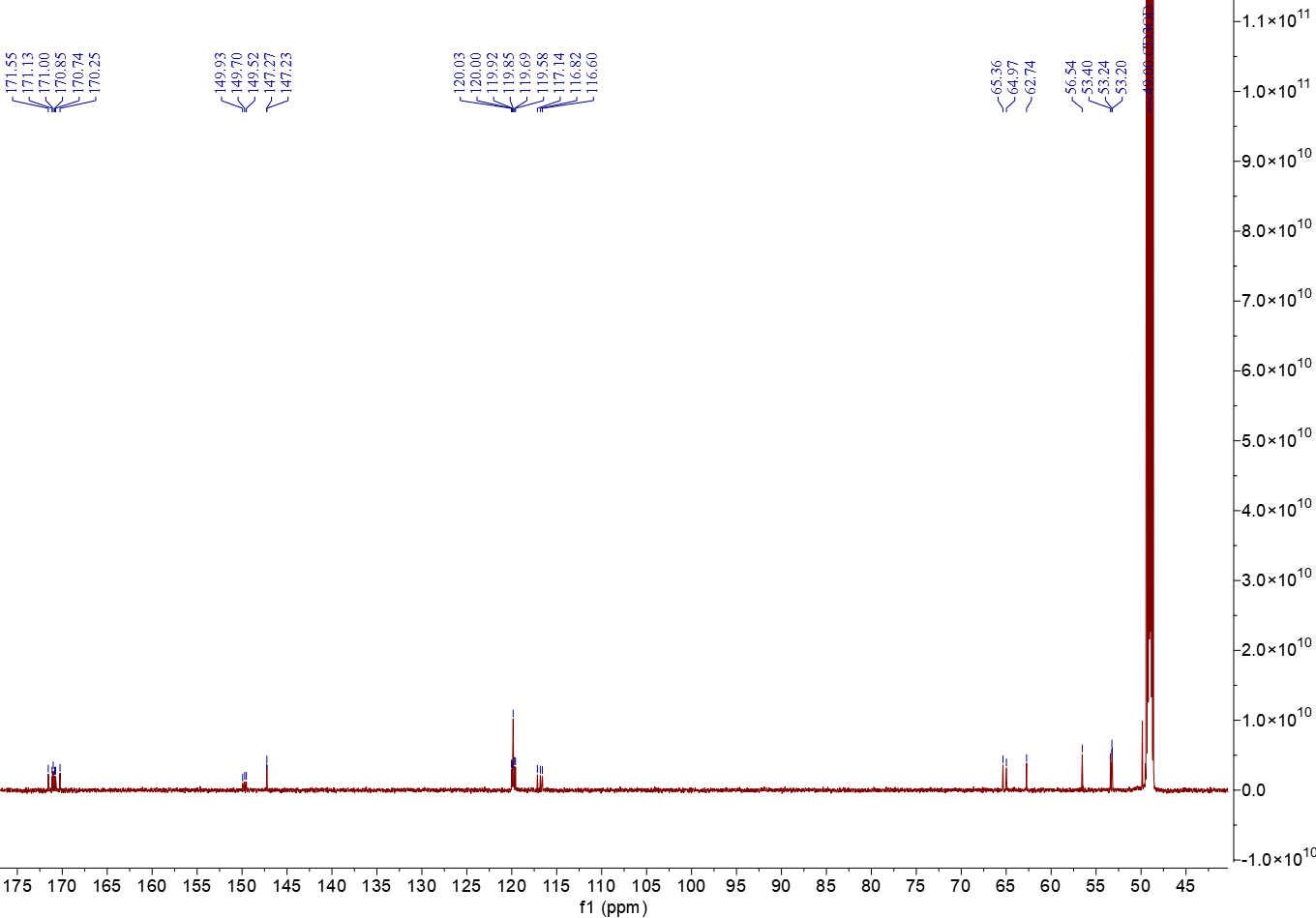


**Supplementary Figure 10.** ^13^C NMR spectrum of compound **2** (CD_3_OD, 175 MHz)


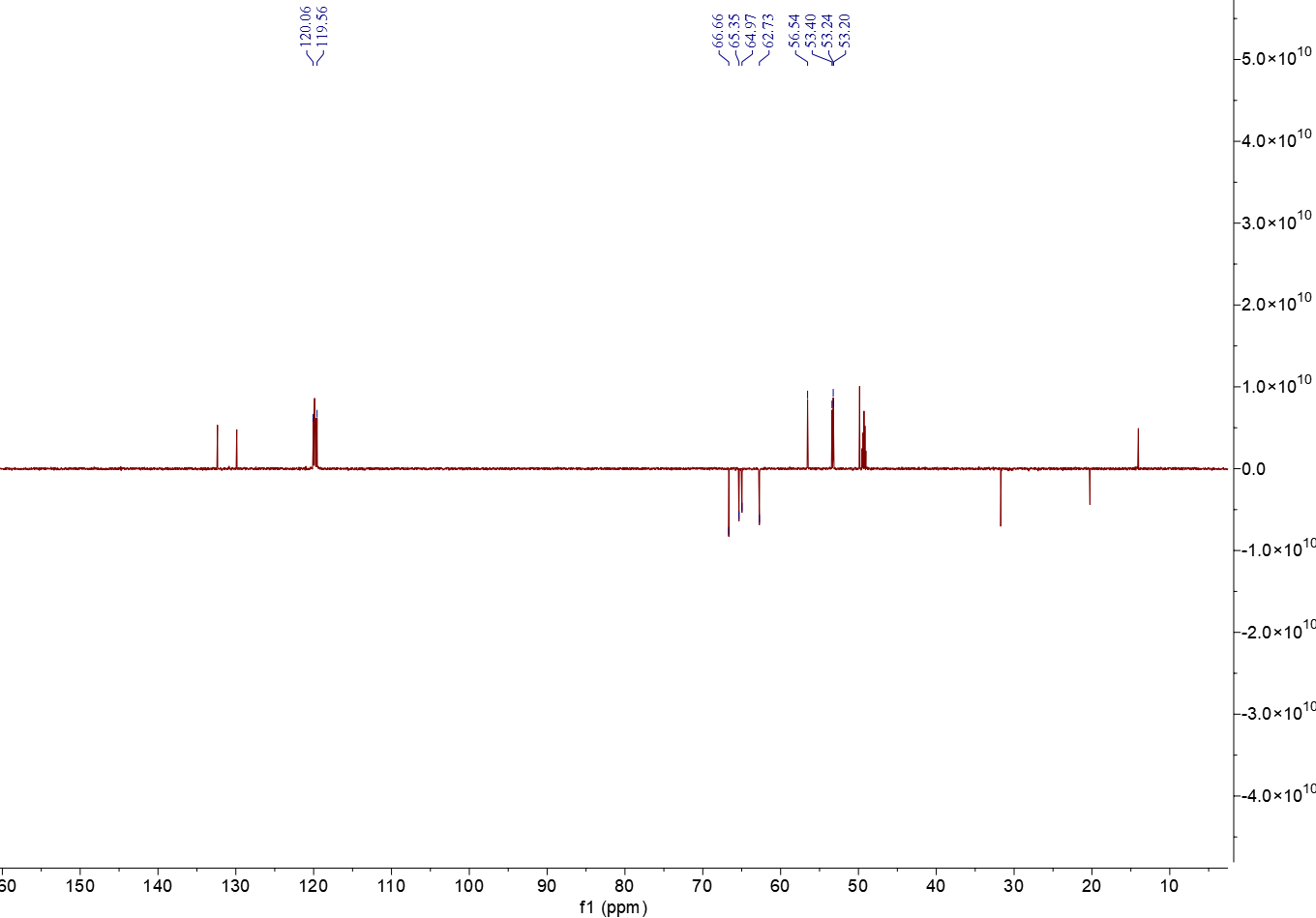


**Supplementary Figure 11.** DEPT135 spectrum of compound **2**.


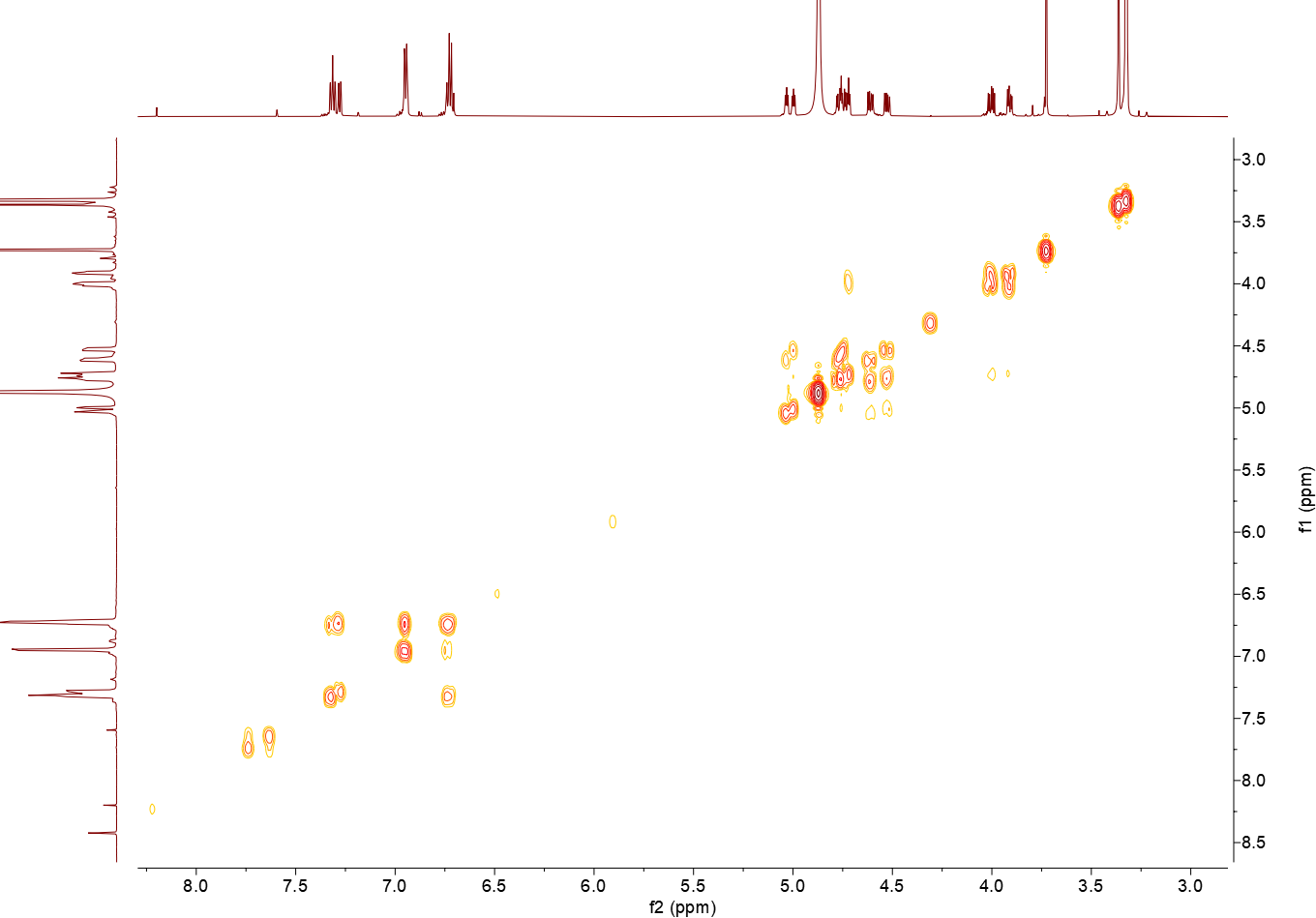


**Supplementary Figure 12.** ^1^H-^1^H COSY spectrum of compound **2**.


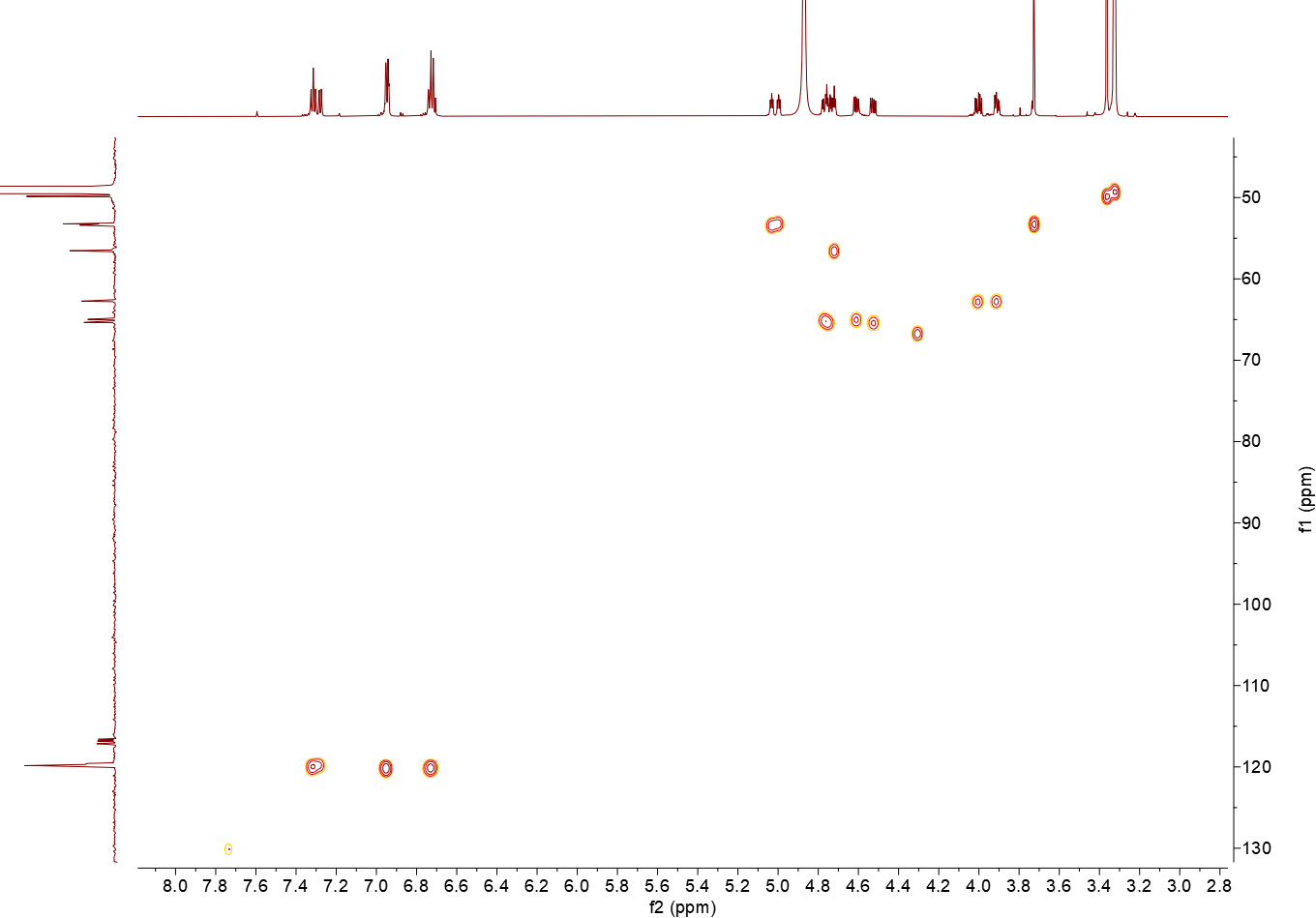


**Supplementary Figure 13.** ^1^H–^13^C HSQC spectrum of compound **2**.


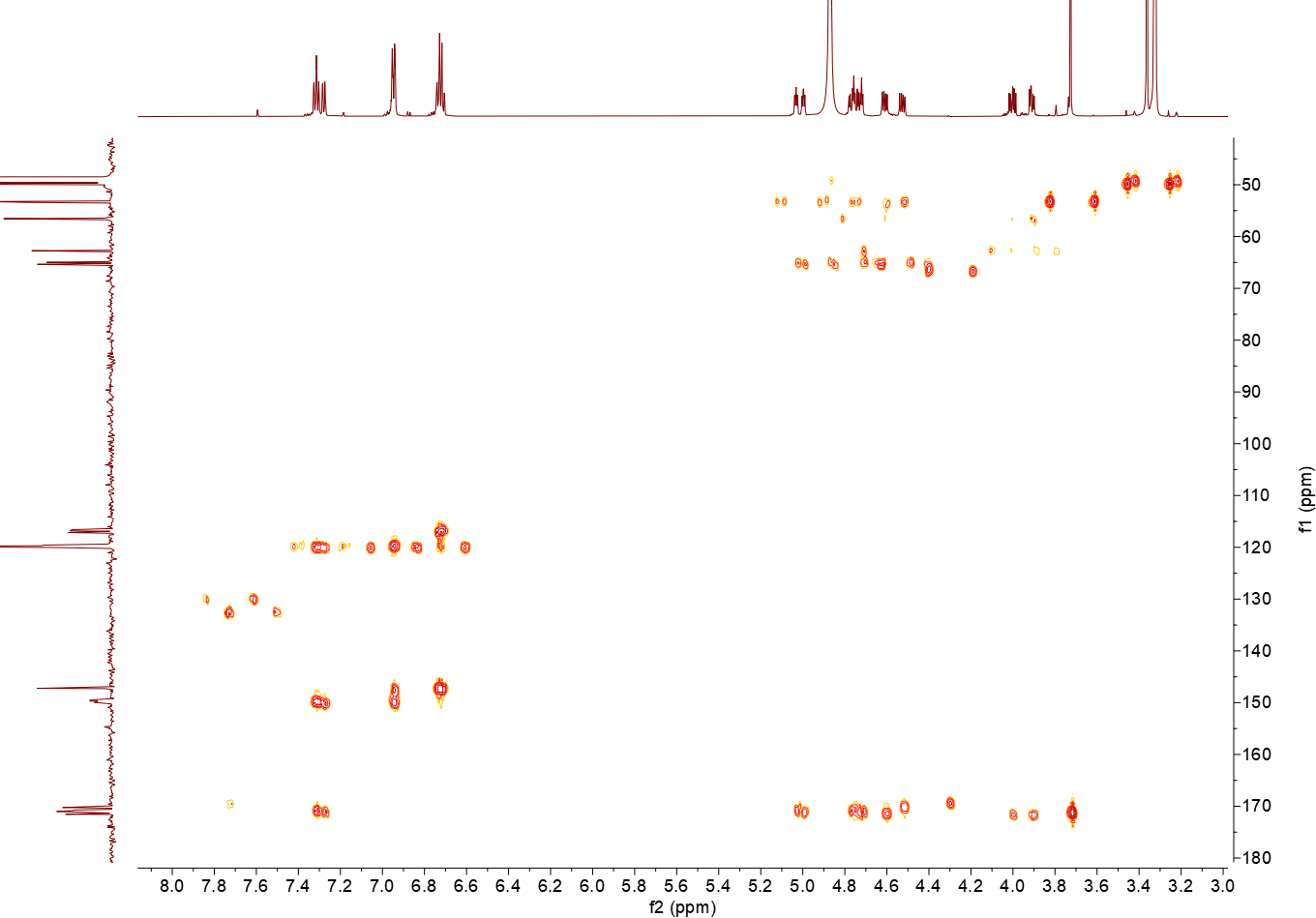


**Supplementary Figure 14.** ^1^H–^13^C HMBC spectrum of compound **2**.


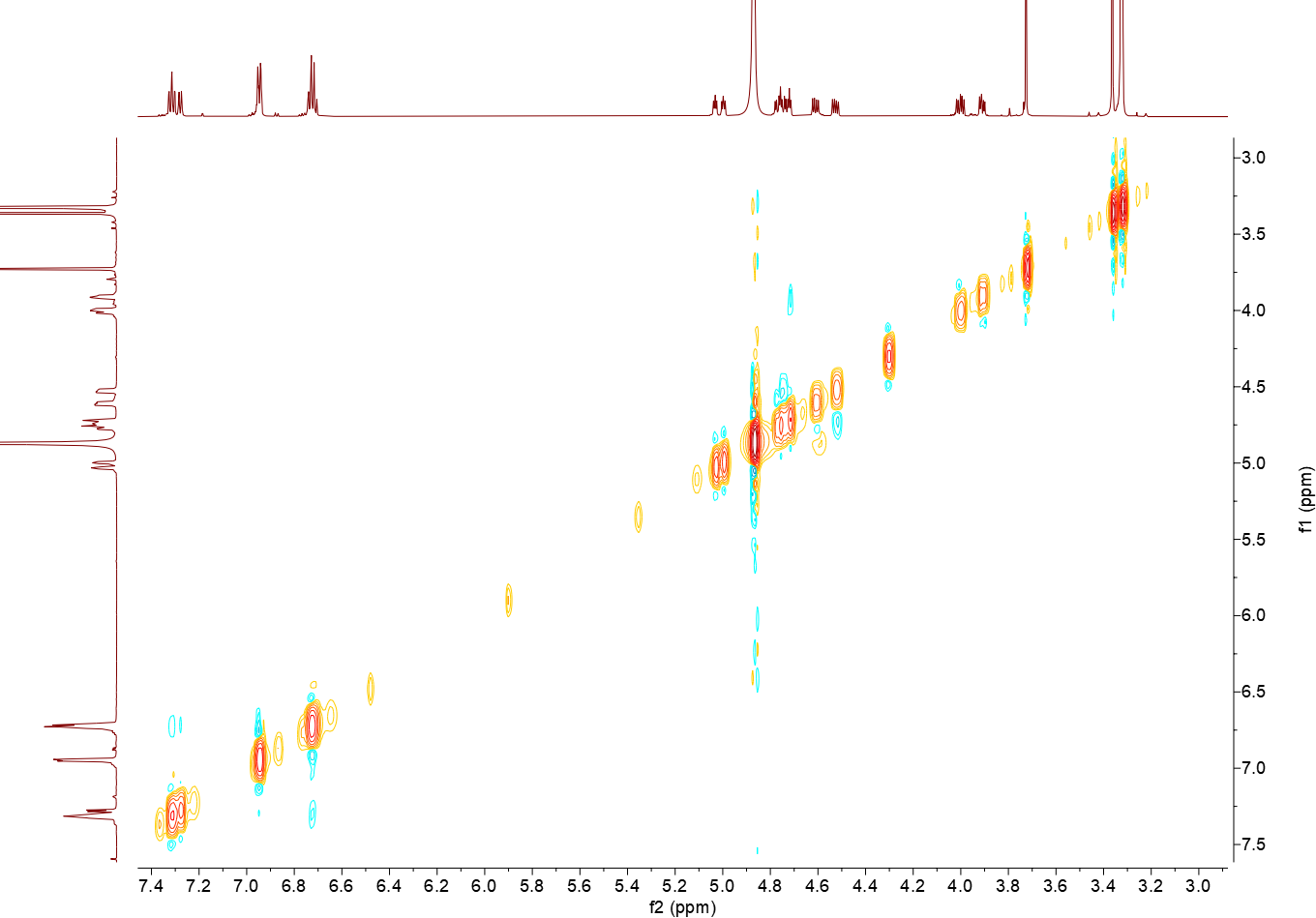


**Supplementary Figure 15.** NOESY spectrum of compound **2**.


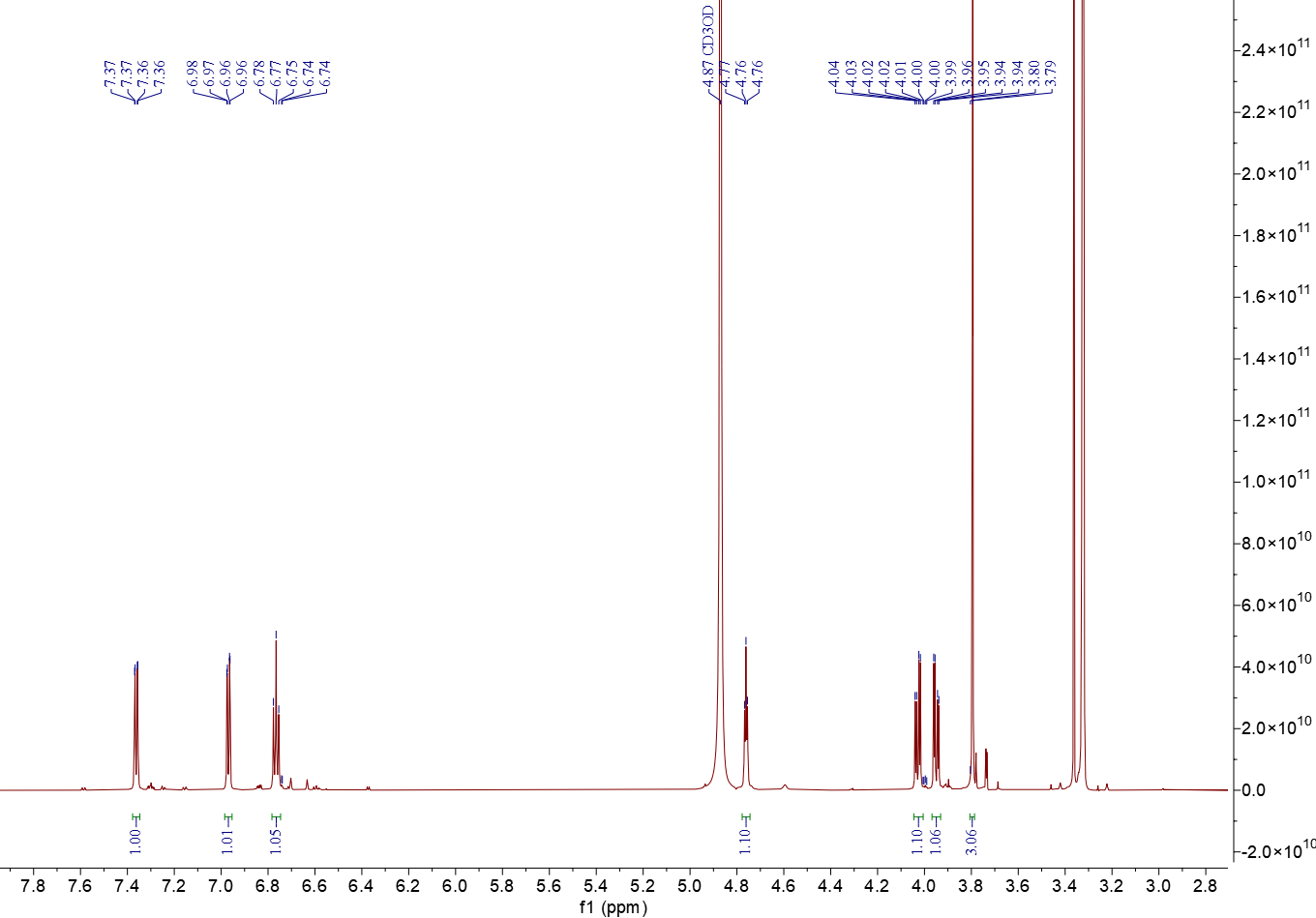


**Supplementary Figure 16.** ^1^H NMR spectrum of compound **3** (CD_3_OD, 700 MHz).


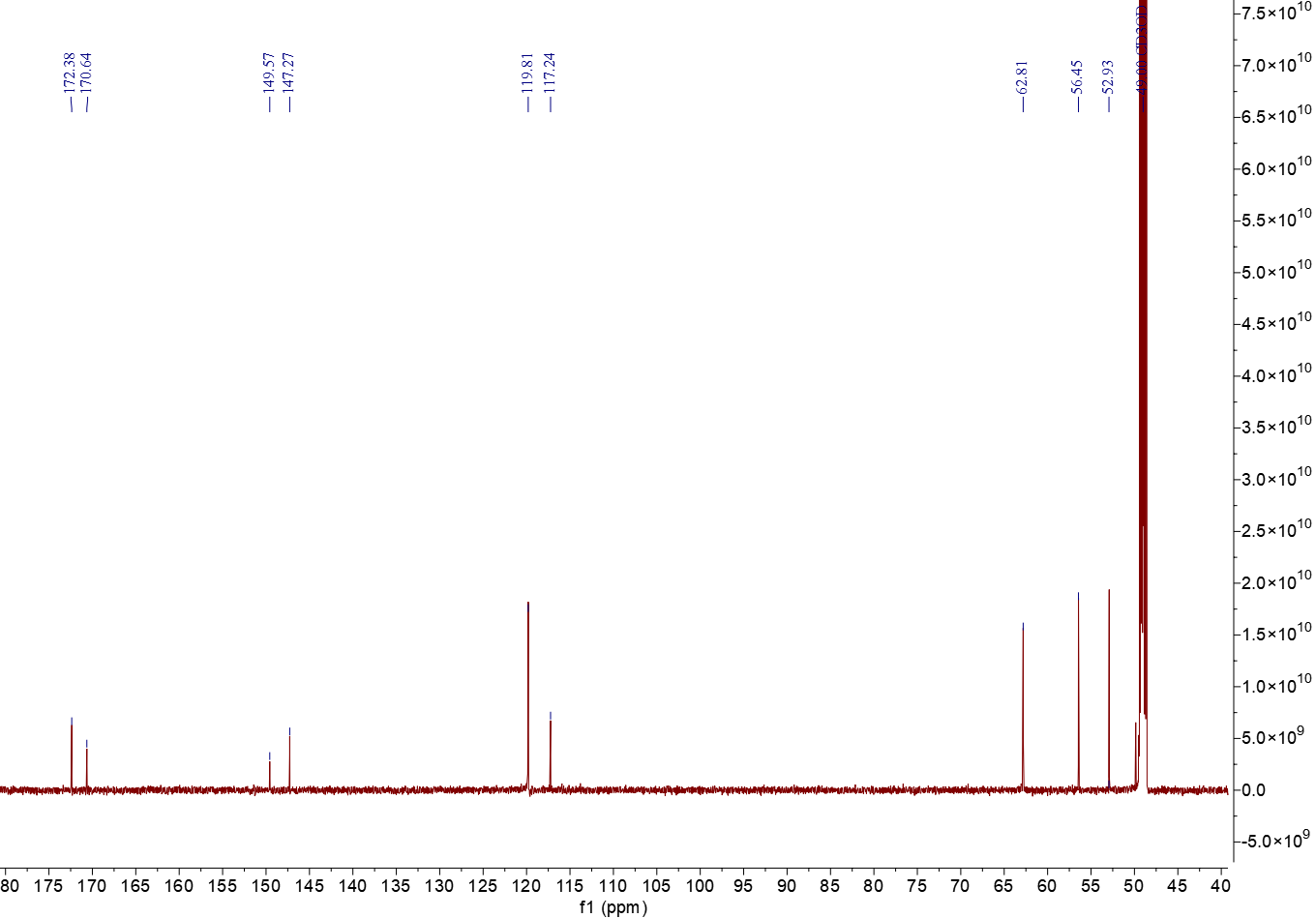


**Supplementary Figure 17.** ^13^C NMR spectrum of compound **3** (D_3_OD, 175 MHz).


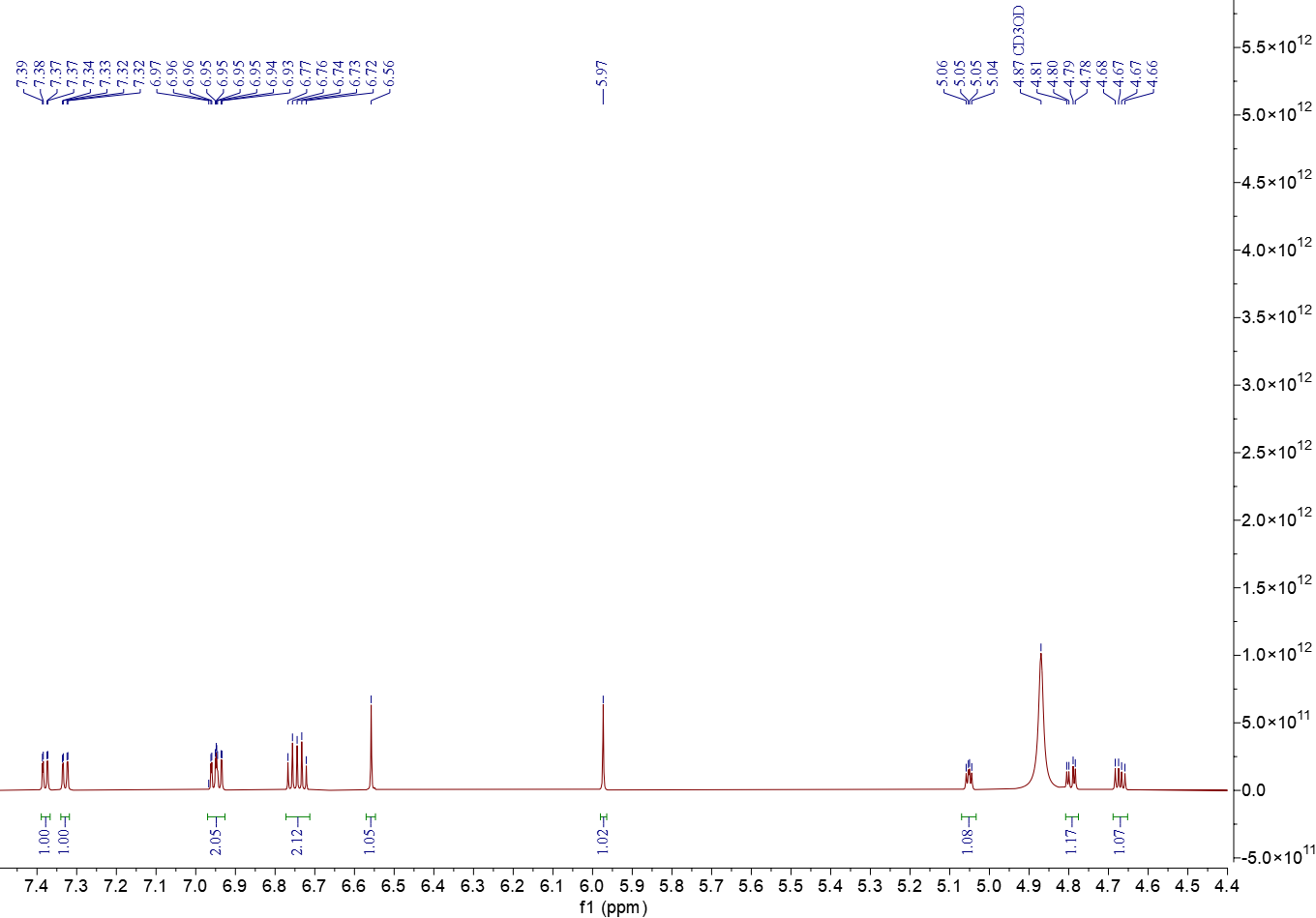


**Supplementary Figure 18.** ^1^H NMR spectrum of compound **4** (CD_3_OD, 700 MHz).


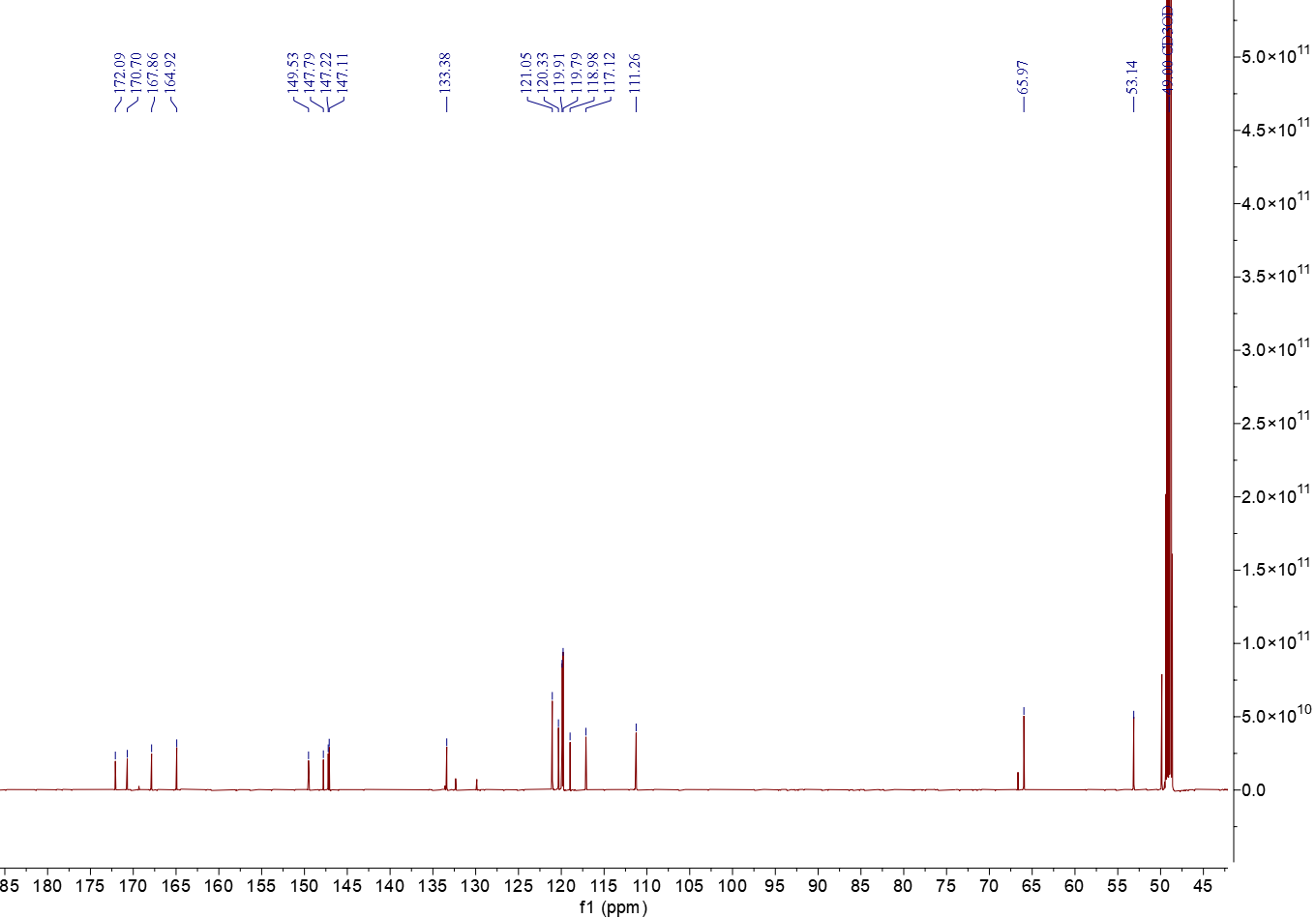


**Supplementary Figure 19.** ^13^C NMR spectrum of compound **4** (CD_3_OD, 175 MHz).


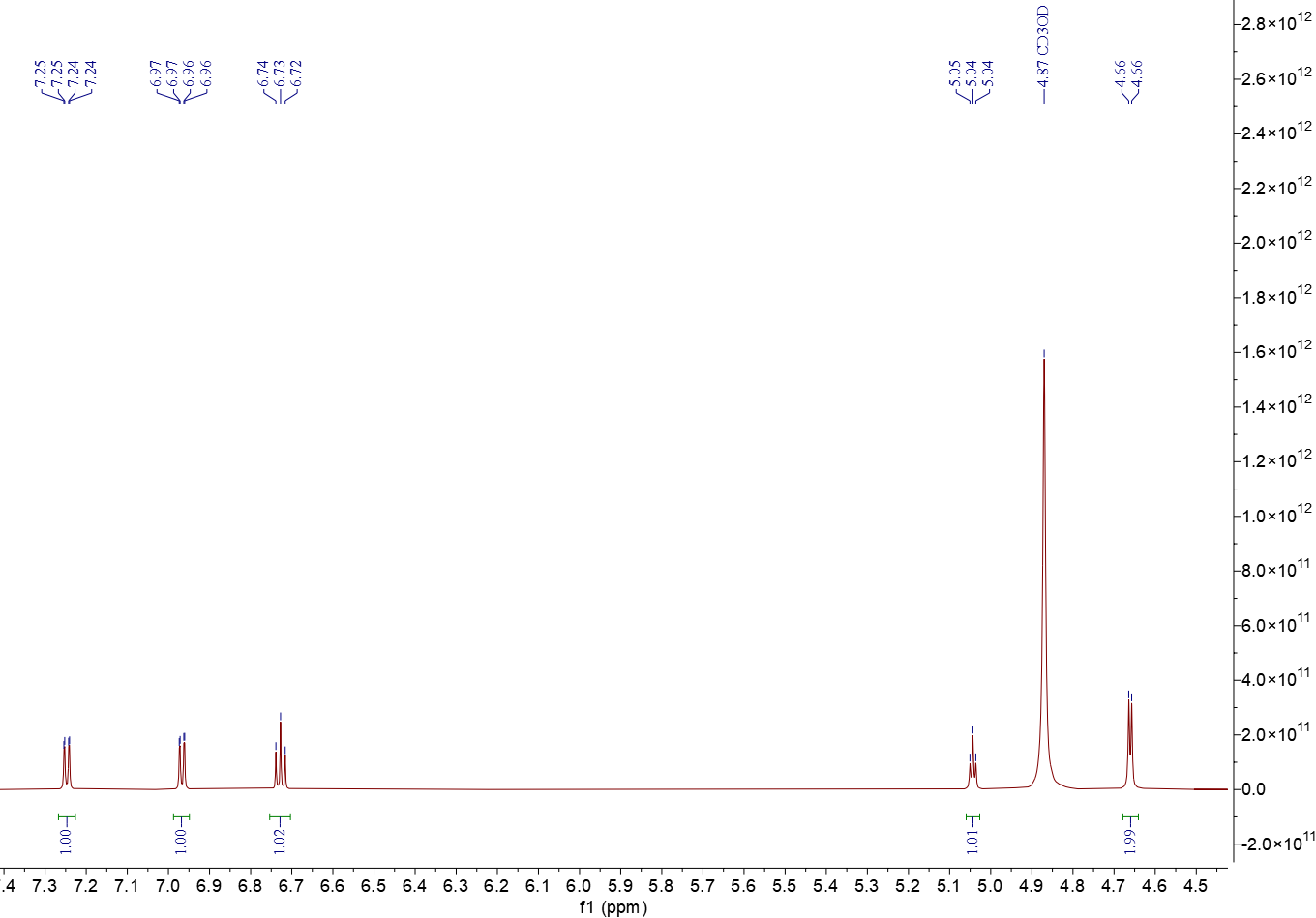


**Supplementary Figure 20.**^1^H NMR spectrum of compound **5** (CD_3_OD, 175 MHz).


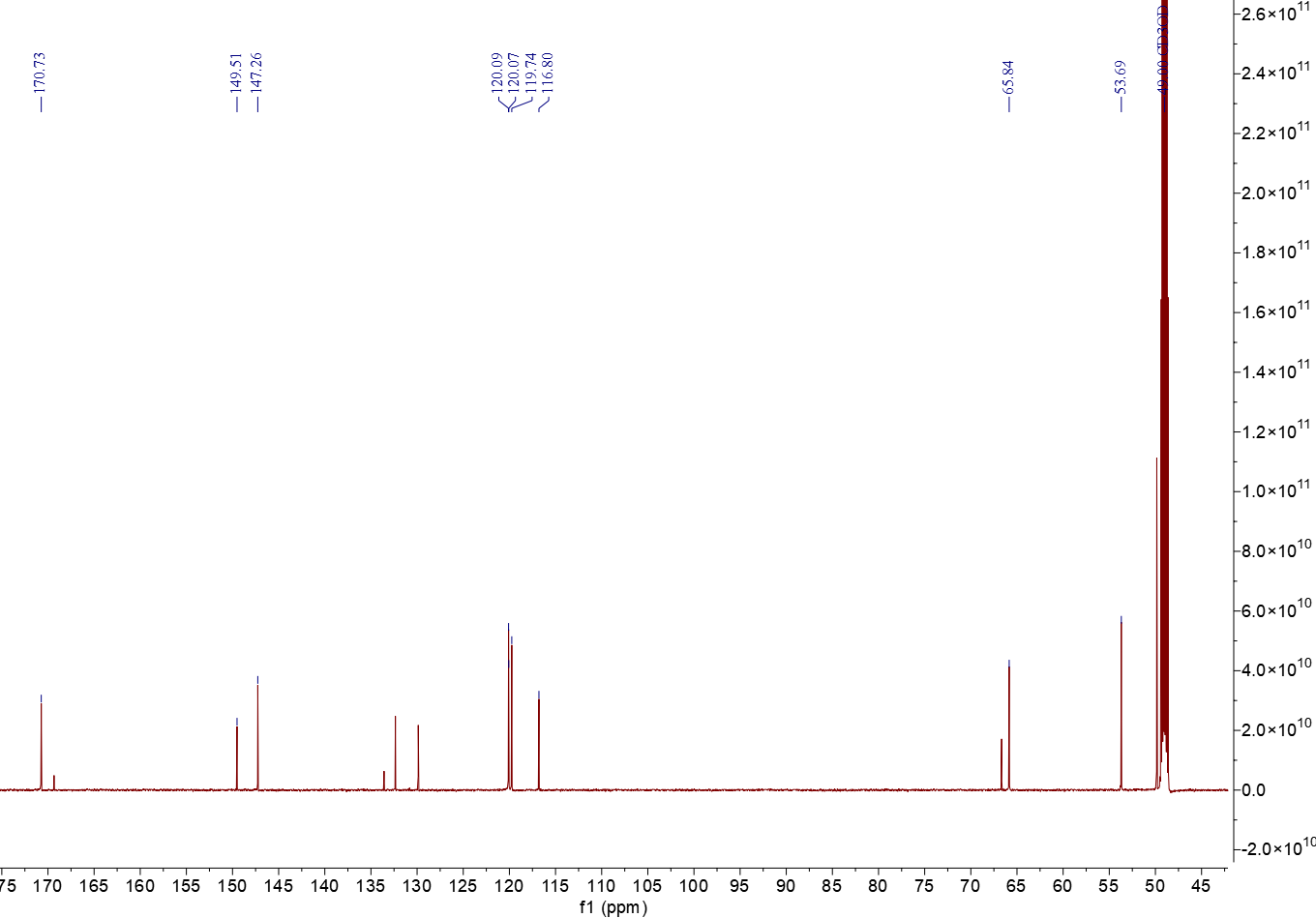


**Supplementary Figure 21.**^13^C NMR spectrum of compound **5** (CD_3_OD, 175 MHz).

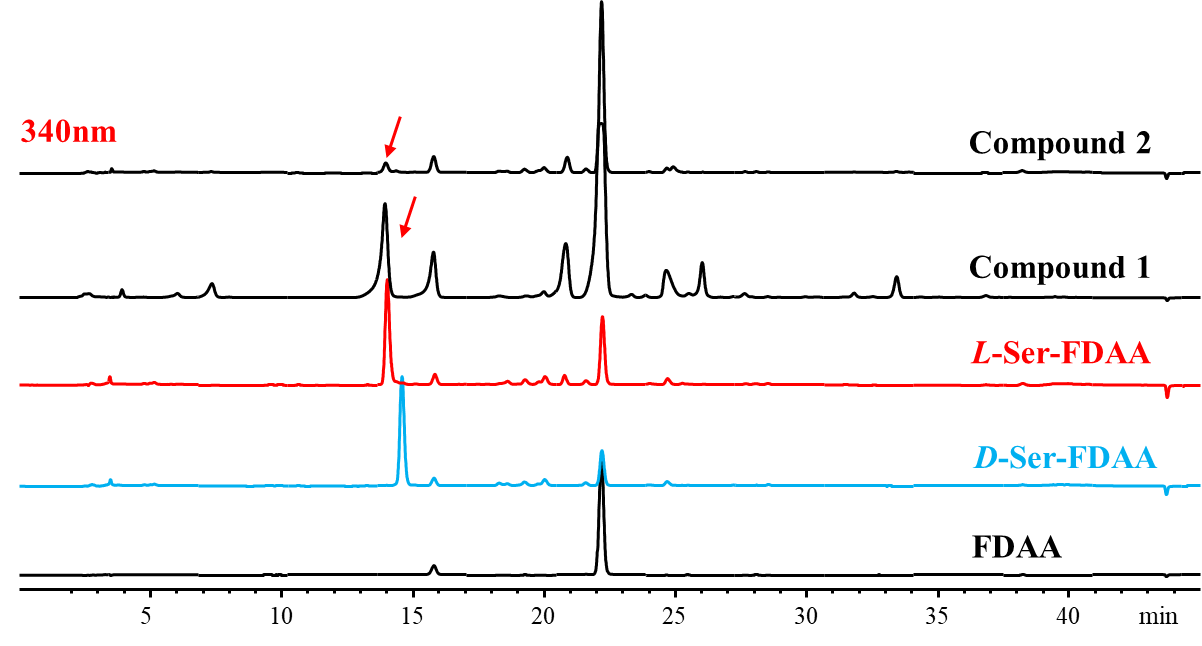


**Supplementary Figure 22.** Marfey's analysis of compound **1** and **2**.

The hydrolysate of Compound 1 and 2 were analyzed by HPLC using *L* -Ser-FDAA (14.0 min) and *D*-Ser-FDAA (14.6 min) as standard.


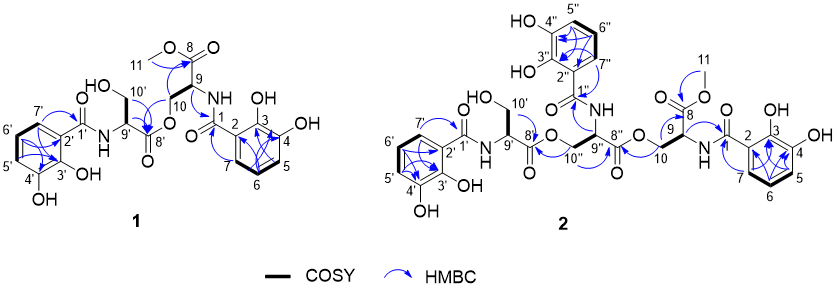


**Supplementary Figure 23**. H−^1^H COSY () and key HMBC () correlations of compound **1** and **2**.

**
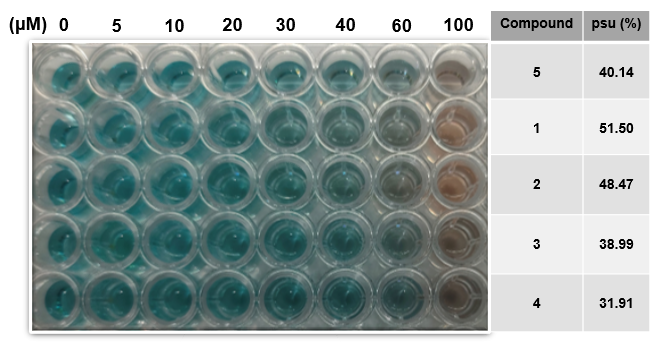
**

**Supplementary Figure 24**. Detection of the siderophore chelating activity of compound **1**-**5** by CAS assay.

Changes in color from blue to orange depend on the concentration of the compound in each well. The amount of siderophore type compounds was expressed in percent siderophore unit (psu) and calculated according to the following formula: psu % = (Ar-As)/Ar ×100. Ar is the absorbance of the CAS solution, as is the absorbance of the CAS solution mixed with the sample containing different concentrations of compounds.

**1.2 Supplementary tables**

**Supplementary Table** S1. Annotation of Enterobactin gene cluster (*ens*) from *S. varsoviensis*.

| Deduced proteins | Size/aa^a^ | Protein homologs^b^ | Proposed function | Identity%/ Similarity% |
| --- | --- | --- | --- | --- |
| EnsT1 | 285 | P23878.2,  *E. coli* K12 | FepC, transporter | 46%/63% |
| EnsT2 | 375 | P23877.2  *E. coli* K12 | FepG, transporter | 32%/55% |
| EnsT3 | 367 | P23876.1  *E. coli* K12 | FepD, transporter | 41%/58% |
| EnsT4 | 327 | P0AEL6.1  *E. coli* K12 | FepB, transporter | 27%/45% |
| EnsA | 259 | P15047.1  *E. coli* K12 | entA, 2,3-dihydro-2,3-dihydroxybenzoate dehydrogenase | 45%/58% |
| EnsC | 523 | P0AEJ2.1  *E. coli* K12 | entC, isochorismate mutase | 40%/55% |
| EnsE | 576 | P10378.3  *E. coli* K12 | entE, 2,3-dihydroxybenzoate-AMP synthase | 48%/65% |
| EnsB1 | 210 | P0ADI6.1  *E. coli* K12 | entB, isochorismatase | 43%/64% |
| EnsB2 | 76 | P0ADI6.1  *E. coli* K12 | entB, PCP | 55%/71% |
| EnsF | 1378 | P11454.3  *E. coli* K12 | entF, enterobactin synthase | 38%/51% |
| U1 | 83 | WP_223769087.1,  *S. huiliensis* | hypothetical protein | 60%/77% |
| U2 | 493 | WP_235483823.1,  *S. roseoverticillatus* | CoA transferase | 72%/78% |
| U3 | 195 | PSJ26611.1, *Streptosporangium nondiastaticum* | RimJ/RimL family protein N-acetyltransferase | 73%/80% |
| G | 420 | [P13039.2](https://www.ncbi.nlm.nih.gov/protein/P13039.2?report=genbank&log$=protalign&blast_rank=3&RID=0),  *E. coli* K12 | Ferric enterobactin esterase | 33%/48% |
| U4 | 672 | WP_221495218.1,  *S. netropsis* | DUF5107(Unknown) | 73%/80% |
| U5 | 362 | WP_251019191.1,  *S.* sp. ISL-11 | Sulfite oxidase | 73%/79% |
| U6 | 266 | WP_110944688.1,  *S. niger* | 2OG-Fe dioxygenase | 74%/82% |
| U7 | 342 | WP_073500609.1,  *Actinacidiphila paucisporea* | [iron-containing redox enzyme family protein](https://blast.ncbi.nlm.nih.gov/Blast.cgi#alnHdr_WP_073500609) | 77%/81% |
| U8 | 102 | WP_268774855.1,  *S.* sp. Je 1-369 | iron-sulfur domain-containing protein | 68%75% |
| EnsM1 | 238 | WP_069462880.1,  *Actinacidiphila rubida* | methyltransferase | 66%79% |
| U9 | 118 | WP_030667203.1,  *S. rimosus* | glyoxalase/dioxygenase | 84%/91% |
| EnsM2 | 269 | WP_181657557.1,  *S. himalayensis* | methyltransferase | 79%/87% |

^a^Numbers are in amino acids.  ^b^Given in parentheses are NCBI accession numbers. Homologues from the enterobactin pathway in *E. coli* K-12 were selected from comparison and highlighted in grey.

**Supplementary Table** S2. Antimicrobial activity of **1** and **2**.

| Strains | MIC(μg/mL) | |
| --- | --- | --- |
|  | **1** | **2** |
| *S. enterica* ATCC 14028 | -*^a^* | - |
| *S. dysenteriae* CMCC 51335 | - | - |
| *E. coli* EDL933 | - | - |
| *K. pneumoniae* HS11286 | - | - |
| *A. baumannii* ATCC 19606 | - | - |
| *P. aeruginosa* PAO1 | - | - |
| *E. faecalis* ATCC 51299 | - | - |
| *S. aureus* ATCC 25923 | - | - |
| *L. monocytogenes* AB97021 | - | 25 |

*^a^* MIC ≥ 100 μg/ml
